# Mast cells and endothelial cells mediate interleukin-33 and ST2 responses in distal chronic obstructive pulmonary disease lungs

**DOI:** 10.1093/ajrccm/aamag079

**Published:** 2026-03-23

**Authors:** Cecilia K Andersson, Premkumar Siddhuraj, Jimmie Jönsson, Johan Ahlgren, Caroline Lindö, Josquin A Nys, Ian C Scott, Sandra Lindstedt, Roland Kolbeck, Alison A Humbles, René Lutter, Yanaika S Sabogal Piñeros, René E Jonkers, Ellen Tufvesson, Caroline Sandén, E Suzanne Cohen, Jonas S Erjefält

**Affiliations:** Department of Experimental Medical Science, Lund University, Lund, Sweden; Medetect AB, Lund, Sweden; Department of Experimental Medical Science, Lund University, Lund, Sweden; Medetect AB, Lund, Sweden; Medetect AB, Lund, Sweden; Medetect AB, Lund, Sweden; Bioscience Asthma and Skin Immunity, Research and Early Development, Respiratory and Immunology, BioPharmaceuticals R&D, AstraZeneca, Cambridge, United Kingdom; Translational Science and Experimental Medicine, Research and Early Development, Respiratory and Immunology, BioPharmaceuticals R&D, AstraZeneca, Cambridge, United Kingdom; Thoracic Surgery, Lund University, Skåne University Hospital, Lund, Sweden; Bioscience Asthma and Skin Immunity, Research and Early Development, Respiratory and Immunology, BioPharmaceuticals R&D, AstraZeneca, Cambridge, United Kingdom; Bioscience Asthma and Skin Immunity, Research and Early Development, Respiratory and Immunology, BioPharmaceuticals R&D, AstraZeneca, Cambridge, United Kingdom; Respiratory Medicine and Experimental Immunology, Amsterdam UMC, University of Amsterdam (formerly Academic Medical Centre of the University of Amsterdam), Amsterdam, The Netherlands; Respiratory Medicine and Experimental Immunology, Amsterdam UMC, University of Amsterdam (formerly Academic Medical Centre of the University of Amsterdam), Amsterdam, The Netherlands; Respiratory Medicine and Experimental Immunology, Amsterdam UMC, University of Amsterdam (formerly Academic Medical Centre of the University of Amsterdam), Amsterdam, The Netherlands; Allergology and Respiratory Medicine, Lund University, Skåne University Hospital, Lund, Sweden; Department of Experimental Medical Science, Lund University, Lund, Sweden; Medetect AB, Lund, Sweden; Bioscience Asthma and Skin Immunity, Research and Early Development, Respiratory and Immunology, BioPharmaceuticals R&D, AstraZeneca, Cambridge, United Kingdom; Department of Experimental Medical Science, Lund University, Lund, Sweden; Allergology and Respiratory Medicine, Lund University, Skåne University Hospital, Lund, Sweden

**Keywords:** COPD immunopathology, IL-33, ST2 splice variants, mast cells, endothelial cells

## Abstract

**Rationale:**

Information is missing on the tissue cell expression patterns of interleukin 33 (IL-33) and splice variants of the IL-33 receptor ST2 in normal and chronic obstructive pulmonary disease (COPD) lungs.

**Objectives:**

To characterize the expression patterns of IL-33, the soluble ST2 (sST2) and membrane-bound ST2 (ST2L) splice variants in the poorly studied small airway and distal lung compartments in COPD and controls.

**Methods:**

Surgically excised lung tissue was collected from 38 COPD patients and 21 non-COPD controls. Lung compartment expression of IL-33 and ST2 and key expressing cell types were assessed histologically by combined in situ hybridization and multiplex immunohistochemistry. Expression dynamics of IL-33, ST2L, and sST2 were explored by spatially resolved single-cell analysis.

**Measurements and Main Results:**

COPD lungs displayed increased IL-33 mRNA and IL-33 mRNA/protein ratios, suggesting increased IL-33 turnover. Total ST2/*IL1RL1* mRNA levels were upregulated in COPD lungs. Mast cells constituted the major ST2-expressing immune cell population in controls and displayed a microenvironmental-specific upregulation of both ST2L and sST2 in COPD. In control alveolar regions, ST2L^high^ sST2^high^ mast cells were present alongside IL-33–expressing general capillary (gCap) and sST2^moderate^ ST2L^low^ aerocyte endothelial subsets. In COPD, patchy alveolar regions displayed markedly elevated capillary sST2 and numbers of ST2L^+^ and IL-33^+^ gCaps.

**Conclusions:**

By unraveling the expression patterns of IL-33 and the biologically opposing ST2L and sST2 splice variants in control and COPD lungs, the present study provides novel insights into IL-33–mediated immunity in the distal lung, information that has bearing on treatment strategies targeting this pathway in lung diseases.

**At a Glance Commentary** 

**Scientific Knowledge of the Subject:** 

Interleukin-33 (IL-33) is a key pro-inflammatory alarmin implicated in chronic obstructive pulmonary disease (COPD) and other inflammatory lung diseases. IL-33 signals through the ST2 receptor, which exists as a membrane-bound signaling form (ST2L) and a soluble decoy receptor (sST2). Although IL-33 and ST2 have emerged as promising therapeutic targets, their spatial expression patterns, cellular sources, and splice-variant dynamics within the distal human lung remain incompletely understood. In particular, how IL-33 and the ST2 splice variants are distributed among immune and structural cell types in COPD has not been defined.

**This Study Adds to the Field:** 

Using multiplex in situ hybridization and spatially resolved single-cell analyses, this study provides the first comprehensive mapping of IL-33, ST2L, and sST2 expression across distal lung compartments in COPD and control lungs. We identify mast cells and specialized alveolar endothelial cell subsets as principal contributors to IL-33/ST2 signaling, revealing microenvironment-specific alterations in COPD. Notably, COPD lungs show increased IL-33 turnover, expanded ST2-expressing mast cell populations, and disease-associated changes in alveolar endothelial subset IL-33/ST2 expression. These findings clarify the cellular architecture of IL-33–mediated immunity in the distal lung and offer mechanistic insight relevant to therapies targeting the IL-33/ST2 pathway.

Impact Statement This study reveals important new insights into the spatial and cellular dynamics of IL-33 and ST2 splice variants in distal COPD lungs, highlighting mast cells and specialized alveolar endothelial cells as key regulatory elements. The findings enhance our fundamental understanding of COPD immunopathology and provide critical mechanistic insights that may refine therapeutic approaches targeting IL-33/ST2 signaling.

## Introduction

Interleukin 33 (IL-33) is an alarmin that initiates inflammatory responses.^1^^,^^2^ The IL-33 protein is prestored in nuclei of structural cells, an arrangement that enables rapid release upon environmental triggers, cell stress, and necrosis. The downstream proinflammatory effects are mediated by cells expressing the membrane variant of IL-33 receptor serum-stimulation-2 (ST2L).^1-3^ Because of its high constitutive expression in the airway epithelium, IL-33 has gained attention as an epithelial-derived alarmin initiating critical immunopathological responses in respiratory diseases.^4-6^ As a result, multiple approaches to pharmacologically target IL-33 or ST2 are in clinical development for indications like asthma and chronic obstructive pulmonary disease (COPD).^7-12^ In COPD, targeting IL-33 by an anti-IL-33 monoclonal antibody, itepekimab, reduced exacerbation rates and improved lung function in patients with a history of smoking.^9^ More thorough assessment of the clinical efficacy and immunological effects of targeting IL-33 or ST2 in COPD is currently explored in several ongoing, or recently completed, phase 3 clinical studies (ClinicalTrials.gov IDs: NCT05166889, NCT05166889, NCT04751487, NCT05326412, NCT05595642).

Despite the extensive in vitro and animal data on lung IL-33 responses and intense clinical trial research, surprisingly, information is missing on the dynamics and expression patterns of IL-33 and ST2 in COPD patient lungs, and even nondiseased human lungs. It is clear that IL-33 is expressed by cell types other than airway epithelial cells.^13^ However, which cell types expressing IL-33 are most prominent or altered in COPD have not been identified. Furthermore, although ST2 was originally identified on Th2 cells, ST2 expression (or signaling upon IL-33 stimulation in vitro) has been shown for a variety of immune cells like ILC2, invariant natural killer T cells, mast cells, eosinophils, basophils, dendritic cells, Th9, and natural killer cells.^1-3^ Some studies have shown expression of ST2 in structural cells such as epithelial cells^14-17^; however, limited response to IL-33 has been demonstrated in vitro.^18^ Endothelial cells may also express both IL-33 and ST2,^19-21^ though the implications for COPD are not yet understood. To get further insight into the regulation of IL-33–mediated responses, it is important to differentiate between the 2 splice variants of ST2: membrane-bound ST2 (ST2L) initiating proinflammatory responses in immune cells, and the soluble form (sST2) thought to act as an anti-inflammatory IL-33 decoy receptor.^3^ The interplay and dynamics of IL-33 mRNA/protein, ST2L, and sST2 in COPD remain to be defined. The lack of information is most critical in the distal lung, an anatomical arena of relevance to COPD where the cell types involved in IL-33–mediated immunity have not been properly identified due to lack of tools providing spatially resolved quantitative data on IL-33/ST2 pathway molecules within distinct cell populations.

This study applies cutting-edge multiplex histological methods and spatially resolved single-cell analyses to reveal the anatomical localization, cellular distribution, and expression dynamics of IL-33, sST2, and ST2L across control lung tissue and COPD (Global Initiative for Chronic Obstructive Lung Disease [GOLD] I-IV) severities. Our data provide novel insights into IL-33–mediated inflammation in the distal lung where altered ST2 splice profiles in alveolar mast cells and dynamic expression of IL-33, sST2, and ST2L in specialized alveolar endothelial cells emerges as an important immunopathological feature in COPD.

## Methods

### Patients and lung tissue sampling

This observational cross-sectional study collected surgically excised lung tissue from 59 patients to map major lung compartments. COPD patients (*n* = 38) were divided into GOLD I-III (*n* = 23) and GOLD IV (*n* = 15); smoking and never-smoking controls (*n* = 21) had no history of inflammatory airway disease. Tissue from COPD GOLD I-III patients and controls was obtained during surgery for well-delineated tumors, ensuring samples were distant and minimally affected from any tumor.^22-24^ COPD GOLD IV tissue came from explanted lungs during transplantation.^22-24^ Inclusion criteria for COPD patients included a significant smoking history and defined COPD per GOLD guidelines; exclusions were atopy, allergic disease, or other airway diseases. Controls had no respiratory disease history or recent infection. All procedures received local ethical committee approval, and all patients provided informed consent. Postsurgery, lung tissues were immediately fixed, dehydrated, and embedded into paraffin blocks. For further details on study subjects and tissue sampling, see Table 1 and the Supplementary Materials and Methods.

**Table 1:** Characteristics of the study participants.

Characteristic	Controls	GOLD I-III COPD	GOLD IV COPD	Overall *P* value
**Participants, *n***	21	23	15	
**Sex, male/female**	9/12	13/10	5/10	
**Age, y**	64 (31-86)	69 (51-84)	59 (50-67)^##^	.002
**BMI, kg/m^2^**	27 (15-53)	25 (23-33)	22 (16-32)	.3
**Smoking history, pack-years**	20 (0-51)	40 (10-55)**	34 (12-60)*	.004
**Smoking status**				
**Never**	5	1	0	
**Former**	12	17	15	
**Current**	4	5	0	
**FEV_1_, L**	2.5 (1.6-4.7)	2.0 (1.1-3.3)^###^	0.8 (0.5-0.9)****	<.0001
**FEV_1_, % of predicted**	93 (59-126)	75 (41-99)*	26 (19-37)**** ^###^	<.0001
**FEV_1_/(F)VC, %**	0.7 (0.6-1.0)	0.6 (0.4-0.7)***	0.3 (0.2-0.3)**** ^#^	<.0001
**Inhaled β2 agonists**				
**Short acting (yes/no/unknown)**	2/19/0	3/20/0	8/6/1^a^	
**Long acting (yes/no/unknown)**	0/21/0	10/13/0	11/3/1^a^	
**Inhaled anticholinergics**				
**Short acting (yes/no/unknown)**	0/21/0	0/23/0	3/11/1^a^	
**Long acting (yes/no/unknown)**	0/21/0	9/14/0	11/3/1^a^	
**Corticosteroids**				
**Inhaled (yes/no/unknown)**	0/21/0	7/16/0	12/2/1^a^	
**Oral (yes/no/unknown)**	0/21/0	2/21/0	2/12/1^a^	

Values are median (range) or *n*.

Abbreviations: BMI, body mass index; COPD, chronic obstructive pulmonary disease; FEV_1_, forced expiratory volume in 1 second; (F)VC, (forced) vital capacity; GOLD, Global Initiative for Chronic Obstructive Lung Disease.

* *P* ≤.05,

** <.01,

*** <.001, and

**** <.0001 for comparison to controls, and

# *P* ≤.05,

## <.01, and

### <.001 for comparison between COPD GOLD I-III and GOLD IV with Kruskal–Wallis with Dunn multiple comparison test.

a One patient with unknown medical history.

### Immunohistochemistry (IHC), in situ hybridization (ISH), and multiplex ISH-IHC

Antigen retrieval and single-triple immunohistochemistry procedures were performed using an automated immunohistochemistry robot (Autostainer LINK, Dako/Agilent, Santa Clara, CA, USA) with signal amplification through EnVision detection kit (Agilent)^24-26^ or tyramide-based amplification. The ISH and detection of gene-specific mRNA probes was performed by the RNAscope ISH system (Advanced Cell Diagnostics, Hayward, CA, USA).^23^^,^^24^^,^^27^^,^^28^ For each patient, IL-33 protein and mRNA and ST2 mRNA were quantified in specified anatomical tissue compartments within large sections containing bronchioles, alveolar parenchyma, and lymphoid aggregates from 2 to 3 separate lung regions.

For identification and assessment of ST2 and IL-33–expressing cells, quadruple ISH was integrated with a platform for multiplex (IHC) staining (Additive Multiplex Labelling Cytochemistry, AMLC, Medetect AB, Sweden).^26^ Data on mRNA and cell identification markers across whole sections, predefined anatomical regions, or within nuclei and cell marker–defined single-cell objects were visualized and quantified by computer-based image analysis (Cell Community Viewer, 2023, Medetect AB, QuPath v0.4.3).^26^^,^^29^

Details on the IHC, ISH, and combined ISH-IHC multiplex methodology, technical controls, primary antibodies, and mRNA probes are shown in Table S1 and the Supplementary Materials and Methods.

### Assessment of IL-1RL1 and IL-33 expression patterns from single-cell RNA sequencing data

Analysis of pan *IL1RL1* (ST2) and IL-33 gene expression across cell types in human lungs was also performed using single-cell RNA sequencing (scRNA-seq) datasets from 2 previously published studies^30^^,^^31^ (see Supplementary Materials and Methods).

### Statistical analysis

GraphPad Prism (version 10.0.0, GraphPad Software, Boston, MA, USA) and JMP (version 17, SAS Institute, Cary, NC, 2023) were used for the statistical analyses. For each variable and group, we calculated mean, median, standard error, standard deviation, and interquartile range. We performed the nonparametric Mann–Whitney *U* test for comparison of 2 sample populations and Kruskal–Wallis test followed by the Dunn post hoc test for comparing 2 or more independent sample groups. In addition, we performed the Spearman correlation test between variables; coefficients (r) were considered statistically significant at *P* <.05.

## Results

### Altered localization and mRNA/protein IL-33 ratio in COPD indicate an increased pathway activation

**Figure 1:**
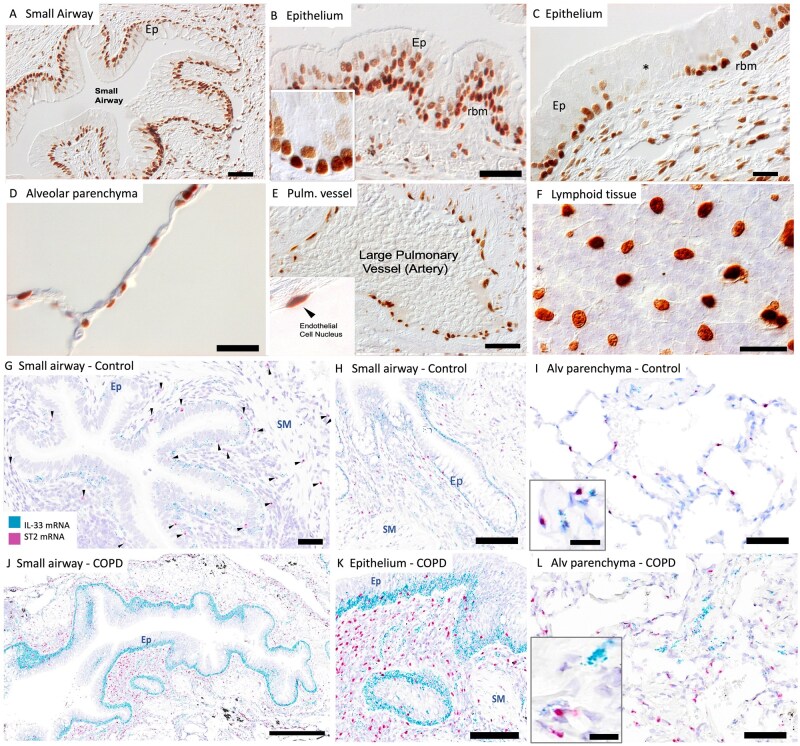
Immunostained IL-33 (brown DAB chromogen) exemplifying the nuclei-stored IL-33 protein distribution patterns across major lung compartments in COPD: (A) small airway/bronchiole (sb = 50 μm), (B) pseudostratified airway epithelium (sb = 50 μm, inset 7 μm), (C) airway epithelium with patchy IL-33 loss (asterisk) (sb = 25 μm), (D) alveolar wall (sb = 20 μm), (E) large pulmonary vessel (sb = 40 μm, inset 7 μm), and (F) lymphoid tissue (sb = 20 μm). (G-L) Representative ISH micrographs exemplifying ST2 and IL-33 mRNA across lung compartments: (G, H) healthy small airway from control subject (sb = 40 μm and 100 μm, respectively), (I) alveolar parenchyma from control patient (sb = 100 μm and inset 20 μm), (J, K) small airway from GOLD IV COPD (sb = 200 μm and 50 μm, respectively) and (L) alveolar parenchyma from COPD lung (sb = 100 μm, inset 15 μm). Black arrowheads point out pan-ST2 mRNA-positive cells. Abbreviations: Alv, alveolar; COPD, chronic obstructive pulmonary disease; Ep, airway epithelium; GOLD, Global Initiative for Chronic Obstructive Lung Disease; ISH, in situ hybridization; rbm, reticular basement membrane; sb, scale bar; SM, smooth muscle.

**Figure 2:**
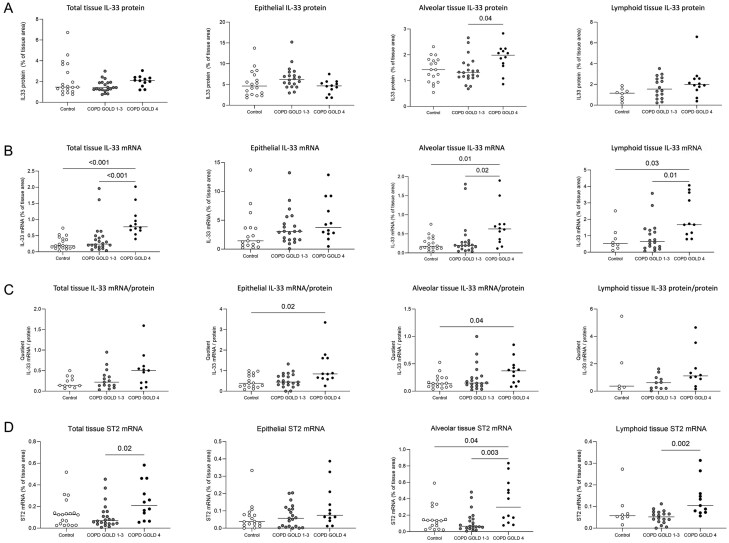
(A) Quantification of immunostained IL-33 protein in control subjects, patients with chronic obstructive pulmonary disease (COPD) Global Initiative for Chronic Obstructive Lung Disease (GOLD) stage I-III and stage IV in total tissue, bronchiolar epithelium, alveolar parenchyma, and ectopic lung lymphoid tissue. (B) Corresponding quantification of IL-33 mRNA marker positivity (as detected by in situ hybridization [ISH]). (C) IL-33 mRNA/protein quotients for total tissue and lung compartments. (D) Quantification of ST2 mRNA (pan-ST2 ISH probe) in controls and COPD patients across lung compartments. Circles denote mean patient values and horizontal bars group medians. Statistical significance between groups was tested using Kruskal–Wallis with Dunn multiple comparison test.

Immunohistochemical assessment revealed that IL-33 protein was restricted to nuclei in structural cells within the small airway epithelium (foremost basal cells), the subepithelial tissue, pulmonary artery and vein endothelium, alveolar septa, and lymphoid tissues (Figure 1A, B; Figure S1 exemplifies the lack of IL-33 across tissue leukocyte populations). Areas of airway epithelium with absent nuclear IL-33, or cytoplasmic extranuclear IL-33 protein, were observed in COPD patients (Figure 1C;  Figure S2). Quantitative image analysis showed that IL-33 protein was increased in the alveolar parenchyma in COPD GOLD IV compared to GOLD I-III patients (Figure 2A). GOLD IV patients had also statistically increased IL-33 mRNA in total lung tissue, alveolar parenchyma, and the lymphoid tissue compared to both milder disease and controls (Figure 2B). The ratio between IL-33 mRNA and protein was higher in GOLD IV COPD compared to controls in the small airway epithelium and alveolar parenchyma (Figure 2C), suggesting an increased IL-33 turnover at these tissue environments. No differences in IL-33 or *IL1RL1* (ST2 isoforms) were observed between inhaled corticosteroids (ICS) users and nonusers in the combined cohort (all *P* >.05) or within COPD alone (all *P* >.05) (Figure S3). ICS dose did not correlate with IL-33 or *IL1RL1* levels.

**Figure 3:**
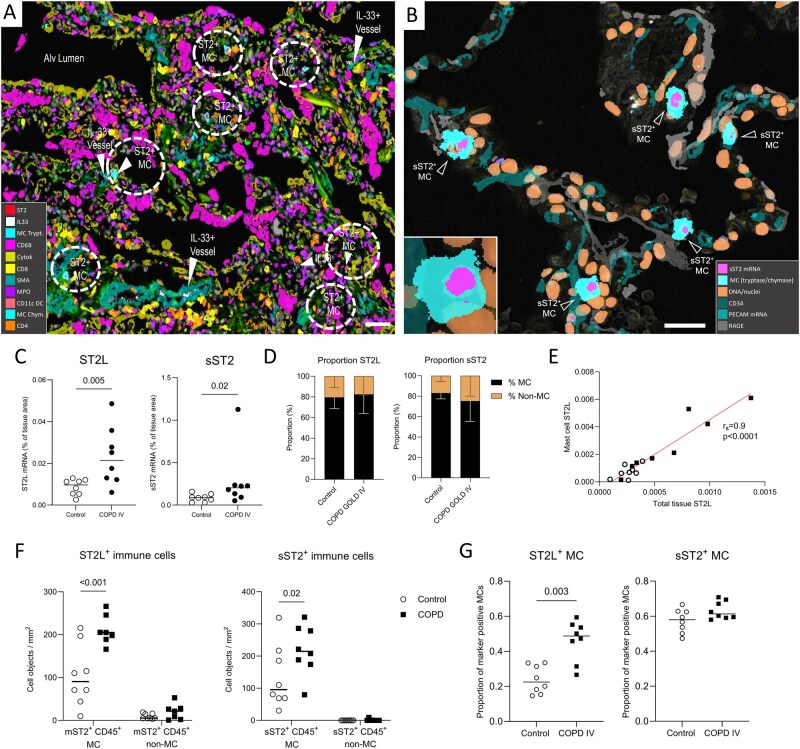
(A) Combined ISH-IHC with pan-ST2 mRNA (bright red), IL-33 mRNA (white), and multiplex IHC imaging with selected pseudocolored markers, including turquoise mast cells. Dotted circles show ST2^+^ MCs and arrowheads denote IL-33^+^ vessels or ST2^+^ MC as stated in the text inside the figure. Scale bar = 60 μm. (B) Visualization of tryptase/chymase-identified mast cells and sST2 mRNA (pink) in the alveolar parenchyma. Arrowheads represent sST2^+^ MCs. Scale bar = 20 μm. (C) Quantitative ISH marker data of tissue expression density (% marker positivity) of ST2L and sST2 mRNA splice variants in total lung tissue from controls and COPD patients. Note the different scales. (D) Total lung tissue proportions of mast cells and non-mast cell with and without ST2L and sST2 ISH mRNA staining. (E) Strong correlation between tissue area fraction of ST2L-positive mast cells and total tissue ST2L mRNA (expressed as ISH probe dots/firm tissue), as investigated with Spearman rank correlation test. (F) Cell density of mast cells and CD45^+^ non-mast cell objects positive for ST2L and sST2 in controls and COPD patients. (G) Data from combined ST2 mRNA ISH detection and multiplex IHC showing the proportion of mast cells (tryptase/chymase) positive for ST2L and sST2 splice variants. Circles denote mean patient values and horizontal bars denote group medians. Statistical significance between groups was tested using Kruskal–Wallis with Dunn multiple comparison test. Correlation levels in (E) were determined by the Spearman test. Abbreviations: COPD, chronic obstructive pulmonary disease; GOLD, Global Initiative for Chronic Obstructive Lung Disease; IHC, immunohistochemistry; ISH, in situ hybridization; MC, mast cell; sST2, soluble ST2; ST2L, membrane-bound ST2.

### Advanced COPD is associated with increased lung tissue expression of ST2/*IL1RL1* mRNA

Pan-ST2 mRNA was detected in the small airway epithelium, the subepithelial lamina propria tissue (Figure 1G, H, J, K). Scattered cells with high ST2 mRNA expression were also found in the alveolar parenchyma (Figure 1I, L). Quantitative ISH analysis revealed increased total tissue, alveolar, and lymphoid tissue expression of pan-ST2 mRNA in advanced COPD compared to patients with COPD GOLD I-III and non-COPD controls (Figure 2D).

### Mast cells are the major sST2- and ST2L-expressing immune cells in control and COPD lungs

To provide insights into differentiated expression and mechanistic insights into potential roles for ST2L and sST2 in COPD, we performed an in-depth and spatially resolved splice variant analysis with combined multiplex ISH-IHC and histology-based single-cell analysis in GOLD IV COPD patients and controls (Table S2). Visualization of ST2 mRNA together with major leukocyte and structural cell markers by multiplex IHC identified mast cells as a major ST2-expressing cell population in both controls and COPD lungs (exemplified in Figure 3A, B). In terms of splice variants, both ST2L and sST2 mRNA increased in COPD lungs (Figure 3C), and histology-based quantitative single-cell analysis showed that mast cell–associated ST2L and sST2 represented the major bulk of the total tissue expression (Figure 3D). A strong correlation was observed between mast cell ST2L and total tissue ST2L (Figure 3E). Further, the numbers of ST2^+^ non-mast cells expressing the pan-leukocyte marker CD45 were dwarfed by the abundant ST2L^+^ and sST2^+^ mast cells (Figure 3F). We also quantified CD45^+^ cells lacking sST2 expression, which were significantly increased in COPD compared with controls (Figure S4). CD45^+^ cells lacking ST2 isoform expression were also significantly increased in COPD lungs (Figure S4), confirming that the expansion of immune cells in COPD is not restricted to ST2^+^ subsets.

**Figure 4:**
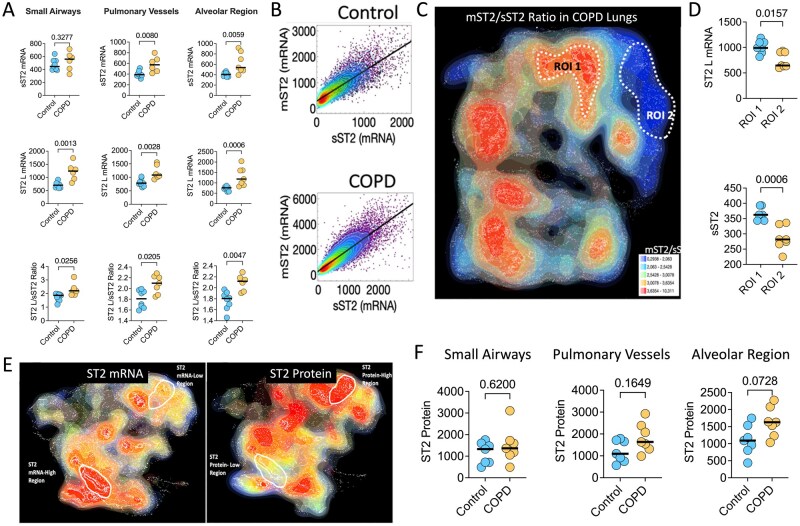
Histology-based single-cell detection of mast cell expression of ST2L and sST2 splice variants. (A) mRNA staining intensity levels of mast cell ST2L and sST2 as well as ST2L/sST2 mRNA quotients across anatomical lung compartments and control vs COPD. Circles denote mean values of >2000 individual mast cells per patient. (B) Correlations between ST2L and sST2 in pooled individual cell data from controls and COPD (GOLD IV). (C) Spatial density map of a large lung section with alveolar parenchymal microenvironments color-coded for regional variations in mean mast cell ST2L/sST2 quotient. The x, y coordinates for individual mast cells are represented by the small white dots. Scale bar = 4 μm. (D) Mean patient expression of ST2L and sST2 in 2 example spatial regions of interest where mast cells display “higher” (ROI-1) or “lower” (ROI-2) mast cell ST2L/sST2 quotients. (E) Paired spatial density maps exemplifying regional variations in mast cell expression of ST2 mRNA (left) and ST2 protein (right). Scale bar = 4 μm. (F) Mean mast cell ST2 protein expression in control and COPD patients (circles denotes mean values/patient of >2000 individual mast cells). Statistical significance between groups was tested using Kruskal–Wallis with Dunn multiple comparison test. Abbreviations: COPD, chronic obstructive pulmonary disease; GOLD, Global Initiative for Chronic Obstructive Lung Disease; ROI, region of interest; sST2, soluble ST2; ST2L, membrane-bound ST2.

### Mast cells in COPD lungs show a spatially complex upregulation of ST2L and sST2

The combined ISH-IHC single-cell approach revealed that the proportion of mast cells positive for ST2L and sST2 increased in COPD (Figure 3G). For both splice variants, the mean level of expression per mast cell (ie, staining intensity) also increased across major lung compartments (Figure 4A). ST2L and sST2 mRNA in mast cells were strongly co-expressed both in control and COPD lungs (Figure 4B). However, the ratio of ST2L over sST2 in mast cells was increased in COPD (Figure 4A; lower panels), albeit spatially resolved single-cell plots revealed a substantial heterogeneity of mast cell ST2L/sST2 ratio across tissue microenvironments (exemplified in Figure 4C, D). This spatial heterogeneity was also observed for mast cell ST2 protein expression, which did display a trend toward increase in the alveolar region (Figure 4E, F). Mast cells are typically divided into “mucosal” MCT cells and “connective tissue” MCTC cells. Interestingly, both ST2 mRNA splice variants were higher in the MCTC subtype (Figure S5), a phenotype shown to increase in COPD.^23^

**Figure 5:**
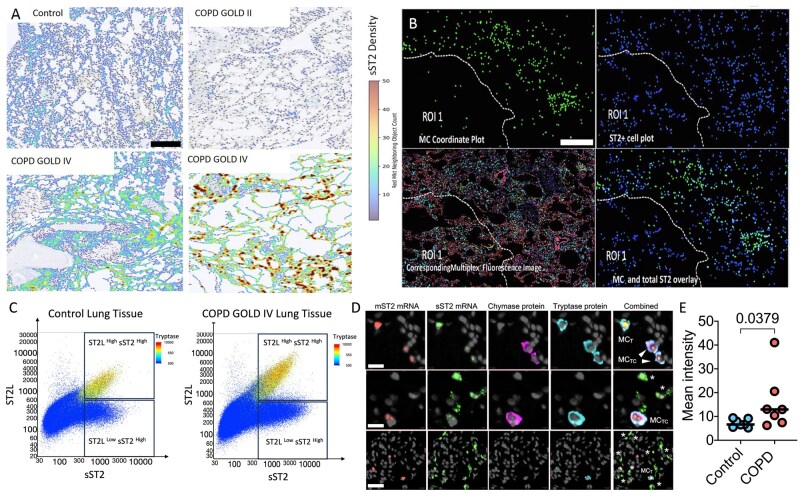
(A) Spatial density maps of sST2 mRNA expression levels in control and COPD lung alveolar tissue. To visualize clustering individual sST2-positive cell objects are marked by dots color-coded for numbers of other sST2 cells within their respective immediate microenvironment. Scale bars = 200 μm. (B) Multiplex combined IHC-ISH staining (down, left) exemplifying an alveolar parenchyma area with a predominance of non-mast cell sST2. The other panels show only x, y coordinates for mast cells and ST2 mRNA-positive cell objects to visualize reveal a region (ROI-1) with local increase in non-mast cells (blue-dots with no mast cell overlap). Scale bar = 200 μm. (C) Histology-based single-cell analysis with sST2 and ST2L expression intensities across >70 000 pooled total alveolar parenchymal cells from controls (left) and COPD lungs (Right). The dots are color-coded for mast cell protease to separate mast cells (light blue/green-yellow/red) from non-mast cells (dark blue). (D) Example images of individual cells where asterisks in the middle and lower panel denote sST2^+^, ST2L-neg non-mast cells. Scale bar for upper and middle panels = 15 μm; lower panel, 40 μm. (E) Quantified mean intensity of sST2 from controls and patients with COPD (individual data for each subject from [C, D] are presented in Figure S6). Statistical significance between groups was tested using Mann–Whitney test. Abbreviations: COPD, chronic obstructive pulmonary disease; GOLD, Global Initiative for Chronic Obstructive Lung Disease; IHC, immunohistochemistry; ISH, in situ hybridization; MC, mast cell; ROI, region of interest; sST2, soluble ST2; ST2L, membrane-bound ST2.

### Identification of alveolar capillary subsets as specialized regulators of IL-33/ST2–mediated immunity in the distal lung

Spatial mRNA density maps revealed patchy distinct alveolar areas with clustered sST2 cell objects in advanced COPD (Figure 5A). In these specific regions, most of the sST2 expression could not be ascribed to mast cells (Figure 5B). Assessment of sST2 and ST2L expression across individual alveolar cells confirmed that, whereas mast cells generally were of an sST2-high, ST2L-high phenotype, the unidentified non-mast cells had a sST2-high, ST2L-low signature and increased in numbers and in COPD (Figure 5C-E and Figure S6).

**Figure 6:**
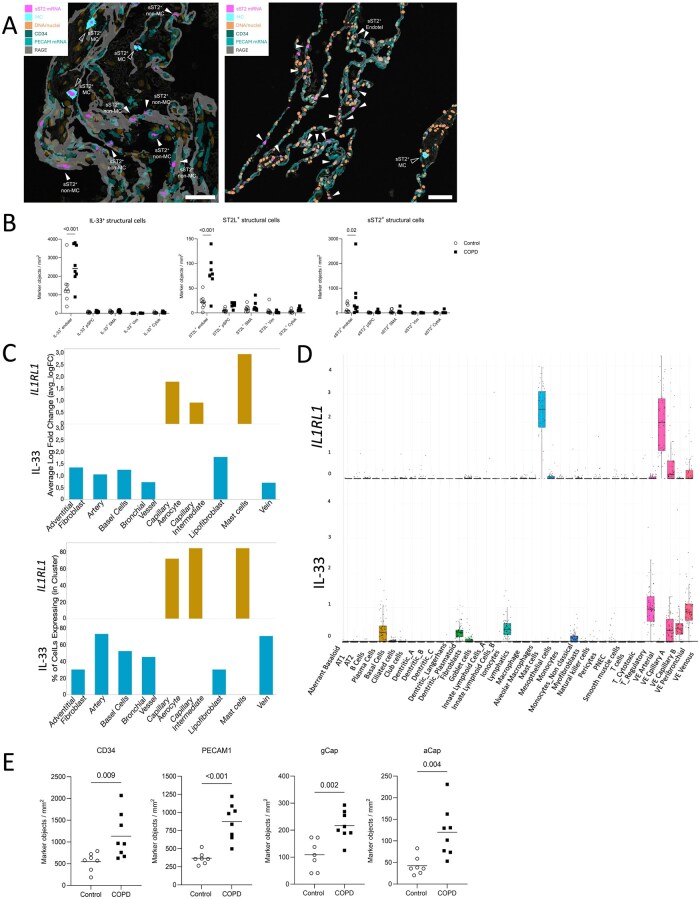
(A) Left: Combined pan-ST2 mRNA (pink), and multiplex IHC imaging with pseudocolored selected markers, including turquoise mast cells. Scale bar = 40 μm. Right: Filled arrows indicate sST2-positive non-mast cell populations and open arrows show sST2-positive mast cells. Scale bar = 50 μm. (B) Quantitative measurements of density of alveolar cell objects positive for IL-33, ST2L, and sST2 mRNA splice across major structural cell populations in controls and COPD lungs (PECAM/CD31^+^ endothelium, pSPC^+^ AT2 pneumocytes, smooth muscle^+^ cells, Vim^+^ fibroblasts, and cytokeratin^+^ AT1 pneumocytes). Single-cell data showing pan-ST2/*IL1RL1* mRNA expression across lung cell populations. (C) Data extracted from a normal lung scRNA-seq dataset showing IL-33 and pan-ST2/IL1RL1 expression across selected cell populations in normal lungs; the expression is shown as average log fold increase (upper panel) and percentage of cells positive for *IL1RL1* or IL-33 (lower panel). (D) scRNA-seq data showing high panST2/*IL1RL1* expression in mast cells and aerocytes (alveolar VE capillary A) whereas gCaps (capillary B) are the IL-33–expressing alveolar capillary cell type (see Supplementary Material for details). (E) Numbers of cell objects positive for CD34 (protein), PECAM1 (mRNA), the gCap marker PTPBR (mRNA), and the aCap marker EDNRB (mRNA) in alveoli of controls and patients with COPD (data are cells per firm alveolar wall tissue area). Statistical significance between groups was tested using Kruskal–Wallis with Dunn multiple comparison test. Abbreviations: aCap, aerocyte capillary; COPD, chronic obstructive pulmonary disease; gCap, general capillary; IHC, immunohistochemistry; scRNA-seq, single-cell RNA sequencing; sST2, soluble ST2; ST2L, membrane-bound ST2; VE, vascular endothelial.

Quantitative combined multiplex ISH-IHC identified CD34^+^, CD31^+^ (PECAM^+^) alveolar endothelial cells as the major non-mast cell ST2 cells; non-mast cell CD45^+^ leukocytes, pro-SPC^+^ type 2 pneumocytes, CD146^+^ pericytes, alveolar smooth muscle, or vimentin^+^ fibroblasts had no or minor expression (Figures 3F, 6A, B). The recently revealed specialized endothelial subsets in alveolar capillaries^30^^,^^32^^,^^33^ motivated a closer look into which endothelial subtypes express IL-33, ST2L, and sST2 and how the expression is altered in COPD. First, to get a crude overview expression pattern, pan-ST2 and IL-33 mRNA profiles were analyzed in publicly available single-cell data sets from dispersed lung cells.^30^^,^^31^ This analysis suggested that in human alveoli, pan-ST2 is primarily expressed in the recently discovered aerocyte capillaries (aCaps) and to a lesser extent in general capillaries (gCaps) (Figure 6C, D). In control lungs the ST2/*IL1RL1* and IL-33 expression seemed mutually exclusive among the alveolar capillary subsets (Figure 6C, D).

**Figure 7:**
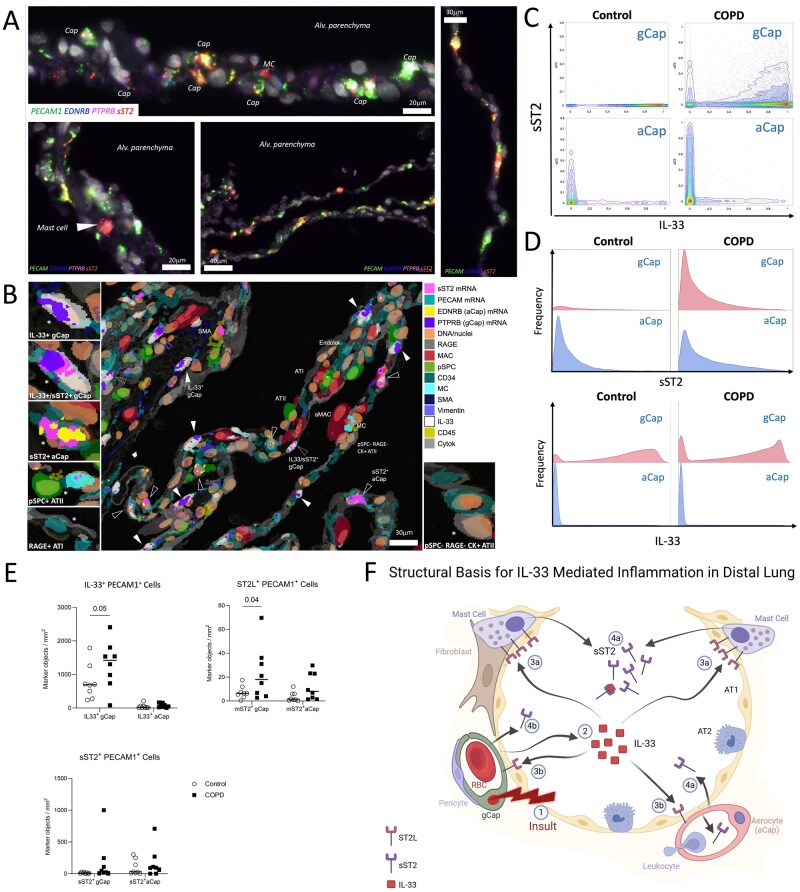
(A) Example of a 4-plex ISH for sST2, CD31/PECAM1, PTPBR, and EDNRB in alveolar walls from a patient with COPD GOLD IV. (B) Combined 4-plex ISH and multiplex IHC imaging with pseudocolored selected markers. Open arrow: sST2^+^ aCap cells; closed arrow: IL-33^+^ gCap cells; dashed arrow: sST2^+^/IL-33^+^ gCap cells. Side panels show high magnifications of exemplifying distinct cell populations positive for sST2, ST2L, or IL-33. Note that due to semi-transparent marker masks, white IL-33 appears light gray. (C) Density scattergrams with pooled group data showing individual cell IL-33 and sST2 expression values for pre-gated PECAM (CD31)/CD34^+^ endothelial cells with either EDNRB^+^ aCap or PTPRB^+^ gCap profiles. (D) Histograms with kernel density curves along increasing IL-33 and sST2 levels for gCaps and aCaps in control and COPD lungs. (E) Quantitative measurements of alveolar tissue density of density of IL-33, ST2L, and sST2 mRNA splice variants in aCap and gCap PECAM/CD31^+^ endothelial populations. Statistical significance between groups was tested using Kruskal–Wallis with Dunn multiple comparison test. (F) Schematic image of a proposed structural basis for IL-33–mediated alveolar inflammation: (1) initial internal or external trigger, (2) release of IL-33 from gCaps, (3a) IL-33/ST2L–mediated mast cell proinflammatory responses, (3b) endothelial ST2L responses, (4a) regulation of acute inflammation by sST2 expressing mast cells and aCaps, (4b) disease-induced patchy upregulation of sST2 also in gCaps. Abbreviations: aCap, aerocyte capillary; Alv, alveolar; Cap, capillary; COPD, chronic obstructive pulmonary disease; gCap, general capillary; GOLD, Global Initiative for Chronic Obstructive Lung Disease; IHC, immunohistochemistry; ISH, in situ hybridization; MC, mast cell; RBC, red blood cell; sST2, soluble ST2; ST2L, membrane-bound ST2.

Next, we performed a combined multiplex ISH-IHC to obtain spatial and quantitative data on endothelial cell densities in alveolar walls and on sST2, ST2L, and IL-33 across aCaps and gCaps (Figures 6E, 7A, B). Utilizing the quantitative strength of simultaneously analyzing spatially close mRNA transcripts for ST2 and capillary subset markers within single cells, we found that in control lungs, IL-33 was restricted to PTPRB^+^ gCaps, while sST2 was mainly expressed in EDNRB^+^ aCaps (Figure 7C, D). In COPD, aCaps retained low IL-33 but showed a modest rise in sST2, whereas gCaps showed more polarized IL-33 expression, displaying both IL-33–negative and IL-33–intense cell populations (Figure 7C, D). Notably, in COPD, gCaps also displayed upregulated sST2 (mean mRNA expression factor 0.0015 in controls and 0.045 in COPD; Figure 7C, D), resulting in appearance of IL-33^+^, sST2^+^ double-positive gCaps (exemplified in Figure 7A, C).

Regarding alveolar endothelial densities, the number of IL-33–positive gCaps per tissue area of alveolar walls increased in COPD, while sST2^+^ gCaps showed a trend toward increase (Figure 7E). Both aCap and gCap had a low baseline expression intensity of ST2L in controls (Figure 7E), with a trend toward increase in COPD (Figure E7). However, in COPD, the numbers of ST2L^+^ gCaps increased (Figure 7E). A similar trend was also seen for ST2L^+^ aCaps. Collectively, these data highlight dynamic alterations of IL-33 and ST2 splice variants across alveolar capillary subsets.

Alveolar ST2 mRNA expression positively correlated with smoking history (pack-years; r_ₛ_ = 0.80, *P* = .005) and showed a negative trend with years since smoking cessation (r_ₛ_ = −0.57, *P* = .05) in COPD GOLD IV patients (Figure S8A). Analysis of IL-33 protein levels revealed that within the COPD cohorts, both total IL-33 and alveolar IL-33 were higher in people who used to smoke compared to people who currently smoke (*P* <.05) (Figure S8B).

## Discussion

In recent years, IL-33–mediated immunity has emerged as a pivotal driver in several respiratory diseases, including COPD. Building on this foundation, our study underscores the distal lung as a critical site for IL-33 activity, detailing both protein and mRNA expression patterns. Importantly, we present the first spatial mapping of the ST2 splice variants ST2L and sST2 in human control lungs and reveal their spatially dynamic upregulation in COPD. Since ST2L and sST2 have contrasting biological roles, our findings provide key insights into how the IL-33/ST2 axis is regulated in COPD lungs. Furthermore, we identify mast cells and specific alveolar endothelial cell subsets as principal mediators of IL-33/ST2-driven responses in the distal lung, shedding new light on potential mechanisms relevant to ongoing clinical programs targeting IL-33 or ST2.^7-12^

Unlike other alarmins like TSLP and IL-25, IL-33 has a high constitutive basal expression and is prestored in structural cell nuclei. The present finding of an elevated IL-33 mRNA/protein ratio in COPD, together with patchy microenvironments with lost or nuclear-cytoplasmic IL-33 translocation, suggests an elevated release and turnover. However, we acknowledge that changes in translational efficiency could also contribute to this observation. Similarly diminished IL-33 staining, alongside increased IL-33 release, was recently demonstrated in viral lower respiratory tract disease.^21^ Our findings also agree with previous reports of elevated IL-33 in sputum, blood, and tissue IL-33 in COPD.^34^ A further indication of an activated IL-33/ST2 axis in COPD was the present increased expression of ST2 in COPD lungs.

The most striking IL-33/ST2 alterations in COPD were found in the alveolar parenchyma, a region left poorly explored in terms of this pathway. Important work on alveolar IL-33–mediated inflammation has been performed in mice.^34^^,^^35^ However, the translational relevance to COPD patients is uncertain since we have previously shown that IL-33 expression in distal mouse lungs is limited to alveolar epithelial type II (ATII) cells.^34^ This is in sharp contrast to human single-cell studies and the present study where both traditional pSPC^+^ ATII cells and distinct pSPC^−^ RAGE^−^, CK^+^, ATII cells, resembling newly identified regenerating ATII cells,^36^ lacked IL-33. Moreover, mice lack alveolar mast cells—highlighted in our study as the key ST2-expressing immune cell.

Although non-mast cell immune cells in COPD lungs may also express ST2L, our data indicate that their numbers, and likely their functional impact, are overshadowed by the abundance of ST2^high^ resident lung mast cells. An alarming-sensing capacity agrees with the general role of mast cells as strategically positioned tissue sentinels and first-line responders to environmental triggers.^37^ Interestingly, both experimental animals and human infants lack alveolar mast cells.^38^ However, a robust resident alveolar mast cell population is present in adulthood.^39^ Indeed, together with alveolar macrophages, mast cells appear to be the major tissue-resident alveolar immune cell population. It remains unknown which mast cell effector functions become activated upon ST2L engagement in COPD. However, several mechanisms of potential relevance to COPD have been reported after IL-33 stimulation of mast cells in vitro. Among these are cytokine and PGD2 release, protease phenotype shift, and enhanced degranulation.^40-43^ Notably, mast cells may also regulate IL-33–evoked inflammation by mast cell protease cleavage of IL-33^44^ and, as supported by this study, increased release of sST2 decoy receptors. It was recently demonstrated that deficiency of mast cell–derived sST2 exaggerates IL-33–driven lung inflammation.^45^ The critical role of mast cells in IL-33–mediated responses in COPD is further supported by the present finding of mast cells increasing both ST2L and sST2 across multiple anatomical regions. Notably, the most noteworthy ST2 increase was in the MCTC type of mast cells, the mast cell phenotype that increases in COPD.^23^

Our findings show that both ST2L/sST2 mRNA levels and ST2 protein/mRNA ratios vary across distinct microenvironments. This suggests that local, yet unidentified, tissue cues dictate the proportion of ST2 splice variants. An example of such tissue-context regulation comes from observations in mice where local production of the prostaglandin E2 drives production of mast cell sST2.^45^ Given our observation of different proportions of ST2 splice variants across mast cells and endothelial cells, exploring the molecular control of the *ILRL1* gene induction and splice variant balance emerges as an important area of research. Some insights into this regulation comes from reports of specific regions within the *IL1RL1* promoter regulating sST2^46^ and novel types of promotor element controlling the *IL1RL1* expression.^47^

IL-33 splice variants play distinct roles in respiratory pathology, with ST2L driving inflammation and airway remodeling while sST2 can dampen immune responses.^13^^,^^15^^,^^48^^,^^49^ We explored whether open lung scRNA-seq datasets could resolve IL-33 and *IL1RL1* (ST2) splice variants at the cell level. The major lung references (eg,^30^) were generated with 10x Genomics 3′ assays, which yield gene-level counts and are not designed to distinguish splice isoforms because the reads typically do not cover the discriminating exons. Consequently, cell type–specific isoform usage cannot be reliably inferred from current open lung scRNA-seq resources. To address this, our study instead employed isoform-specific ISH probes (ST2L vs sST2) combined with multiplex immunohistochemistry. Future work using full-length (Smart-seq3/FLASH-seq) or long-read scRNA-seq (eg, LR-Split-seq) will be required to comprehensively profile IL-33 and ST2 splice variants at single-cell resolution in the distal lung.^50^  *IL1RL1* (ST2) harbors promoter/intronic and coding variants, including TIR-domain haplotypes, that modulate receptor signaling and sST2 levels, and contribute to asthma endotypes. However, robust trans-effects from *IL1RL1* to IL-33 expression in lung have not been reported. Current lung scRNA-seq atlases lack genotype information and cannot resolve *IL1RL1* haplotypes. Although targeted genotyping from our formalin-fixed, paraffin-embedded samples is technically feasible, the present study is underpowered for meaningful genotype–phenotype analyses, particularly for lower-frequency coding variants. Larger, genotype-enabled COPD cohorts will be required to assess whether *IL1RL1* haplotypes influence distal lung IL-33/ST2 pathways.^51^

Using our novel histology-based single-cell analyses, we provide new insights into the IL-33/ST2 axis in the human alveolar microcirculation. Although we did see IL-33 restricted to gCaps and total ST2 predominately in aCaps, our novel splice variant analysis challenge earlier scRNA-seq results,^32^^,^^52^^,^^53^ and our current scRNA-seq analysis, both of which forwarding the concept of IL-33–expressing gCaps and ST2-epxressing aCaps. Specifically, our histology data showed that in healthy lungs, sST2 is predominantly found in aCaps, yet expression also occurs in gCaps—an important nuance missed in standard scRNA-seq, likely due to limitations in detecting isoform-specific transcripts. Further, our histological approach with immediate tissue fixation minimizes the ex vivo proteome and transcriptome alterations occurring during cell preparations for high-plex single-cell approaches.^54^^,^^55^ Our ISH (IL-33 mRNA) and IHC (IL-33 protein) measurements rely on positive-pixel/voxel fractions and are therefore semiquantitative rather than absolute. To minimize sampling and processing bias, we applied standardized fixation times and tissue handling, included internal positive controls for both assays, digitized all slides on the same scanner with locked exposure/illumination settings, and used a predefined, invariant thresholding pipeline across all samples. Even with rigorous controls, minor differences in sectioning and partial-volume mixing cannot be fully excluded; accordingly, we interpret our readouts comparatively (rather than as absolute levels) and note that ISH and IHC display concordant spatial patterns.

Our histology-based data also show that in COPD sST2 is robustly upregulated also in gCaps, accompanied by a discernible rise in the membrane-bound ST2L variant. These novel spatial data underscore a dynamic IL-33/ST2 signaling environment that extends beyond a simple “IL-33^+^ gCap vs ST2^+^ aCap” model. Such plasticity may be highly relevant to disease pathogenesis: Enhanced sST2 released from both aCap and gCap could more efficiently modulate IL-33–mediated inflammation or reparative processes. Likewise, the present expansion of ST2L^+^ gCaps would enhance the proinflammatory impact of IL-33 in inflamed COPD lungs. This notion is supported by recent in vitro observations of IL-33–stimulated endothelial cells upregulate cytokines, chemokines, and adhesion molecules.^21^ Collectively, our findings suggest that, in COPD, alveolar endothelial subsets are more heterogeneous and functionally adaptable than previously appreciated, pointing to a potential therapeutic window in modulating ST2 isoforms to optimize alveolar repair and limit pathological inflammation.

Our data may also have biomarker implications. Since both alveolar capillaries, and mast cells, are in direct physical contact with the pulmonary circulation, it is likely that these populations contribute significantly to the elevated serum sST2 level, which has been forwarded as a biomarker of poor prognosis, not only in COPD but also in other lung conditions like COVID-19 and pulmonary hypertension.^56^^,^^57^

Because of the complexity and resource-intensive nature of the present histological single-cell explorations, a limitation of the present study is the modest patient numbers. This precludes a statistically powered comparison between the present IL-33/ST2 parameters, smoking status, and eosinophil/type 2 signatures. The limited numbers of GOLD stage I-III, most of whom were classified as mild-moderate GOLD I-II, may also explain why fewer statistical alterations in IL-33 and ST2 were found for this less severe category. Thus, this study was not designed to follow up on the reported increased IL-33 levels and clinical response to anti-IL-33 treatment in COPD patients with a smoking history compared to currently smoking patients.^9^^,^^20^ Nevertheless, our findings suggest that accumulated cigarette smoke burden is associated with increased alveolar ST2 and that among people with a history of smoking, the expression may be reduced with time since cessation (Figure S8A). It is also worth mentioning that both total tissue and alveolar IL-33 protein was higher in former compared to current smokers (Figure S8B), indicating that the previously observed phenomenon of higher bronchial IL-33 in COPD patients with a smoking history may extend into distal lung compartments.^20^ Naturally, our cross-sectional design also prevents explorations of the longitudinal plasticity of the IL-33 and ST2 splice variant expression.

Liberated IL-33 rapidly undergoes oxidation^58^ and it has recently been suggested that, besides IL-33/ST2 activation, IL-33 signaling may also be executed by binding of oxidized IL-33 to an receptor for advanced glycation end-products (RAGE) / epidermal growth factor receptor (EGFR) receptor complex on airway epithelial cells.^18^ New data from COVID-19 lungs recently showed that RAGE/EGFR can also be expressed on alveolar type 1 epithelial cells. Hence, although explorations of the RAGE/EGFR complex is outside the scope of the present study, AT1 cells should be regarded as a tentative population that, besides mast cells and capillary subsets, can effectuate IL-33–mediated responses.^18^

In summary, our data shed light on novel facets of alveolar IL-33–mediated immunity in the distal human lung. Baseline IL-33 and ST2 expression patterns support a rapid mast cell and endothelial response to IL-33 release from the IL-33^+^ gCap population; once acute inflammation is triggered, both mast cells and aCaps can curb the response via soluble ST2. In COPD, the IL-33/ST2 axis is upregulated, and gCaps transform into an IL-33–sensing and sST2-producing pool. Additional ST2L^+^ immune cells likely reinforce these proinflammatory pathways in chronic disease. Given IL-33’s broad effector functions, the patchy changes in ST2 and IL-33 expression patterns we describe here may substantially influence spatially complex inflammatory processes and disease progression in COPD. Overall, our findings highlight a nuanced equilibrium between ST2L and IL-33–neutralizing sST2, emphasizing the need for deeper exploration to guide and refine anti-IL-33–based therapeutic interventions across diverse COPD patient groups.

## Supplementary Material

aamag079_Supplementary_Data

## Data Availability

This article has an online data supplement, which is accessible from the Supplements tab.
