# Supplementary material for: Mast cells and endothelial cells mediate interleukin-33 and ST2 responses in distal chronic obstructive pulmonary disease lungs
Source: Am J Respir Crit Care Med. 2026 Mar 23;212(7):1495–509. doi: 10.1093/ajrccm/aamag079 (PMC13318233; doi:10.1093/ajrccm/aamag079)
Supplement: aamag079_Supplementary_Data [file aamag079_supplementary_data.zip › Online Supplement with Figures_clean_5-DEC-2025.pdf]

Online Data Supplement

Mast Cells and Endothelial Cells Mediate Interleukin-33 and ST2 Responses in Distal COPD Lungs

Cecilia K Andersson[1,2], Premkumar Siddhuraj[1], Jimmie Jönsson[2], Johan Ahlgren[2], Caroline Lindö[2], Josquin A Nys[3], Ian C. Scott[4], Sandra Lindstedt[5], Roland Kolbeck[3†], Alison A Humbles[3#], René Lutter[6], Y.S. Sabogal Piñeros[6], René E Jonkers[6], Ellen Tufvesson[7], Caroline Sandén[1,2], E. Suzanne Cohen[3], Jonas S Erjefält [1,7].

[1]Experimental Medical Science, Lund University, Lund, Sweden.

[2]Medetect AB, Lund, Sweden.

[3]Bioscience Asthma and Skin Immunity, Research and Early Development, Respiratory & Immunology, BioPharmaceuticals R&D, AstraZeneca, Cambridge, UK.

[4]Translational Science and Experimental Medicine, Research and Early Development, Respiratory & Immunology, BioPharmaceuticals R&D, AstraZeneca, Cambridge, UK

[5]Thoracic Surgery, Lund University, Skåne University Hospital, Lund, Sweden.

[6]Respiratory Medicine and Experimental Immunology, Amsterdam UMC, University of Amsterdam, Amsterdam, The Netherlands, formerly Academic Medical Centre (AMC) of the University of Amsterdam.

[7]Allergology and Respiratory Medicine, Lund University, Skåne University Hospital, Lund, Sweden.

[†]*Current affiliation: Aclaris Therapeutical, Wayne, PA, USA.*

[#]*Current affiliation: ARC Hub for Therapeutics, Trinity College Dublin, Ireland*

DETAILED MATERIAL AND METHODS

Patient characteristics and tissue sampling

To allow a detail mapping of major lung compartments surgically excised lung tissue was collected as part on an observational cross-sectional study.

In total, 59 patients with chronic obstructive pulmonary disease (COPD) and controls subjects were included in the study. Surgical tissues were collected and processed for histologic analysis as previously described (1, 2). The COPD patients, which ranged from mild to very severe disease, were divided into two categories: GOLD I–III (n=23) and GOLD IV (n=15). Persons with no history or signs of any inflammatory airway disease served as controls (n=21).

The tissue samples from both COPD GOLD I-III and smoking and non-smoking controls were collected from patients undergoing surgery for well-delineated tumors. For the control subjects care was taken to collect peripheral lung tissue as far from the tumor as possible, a procedure that has repeatedly been used to collect lung tissue research samples(3-5). Tissue from COPD GOLD IV were from explanted lungs collected in connection to lung transplantation surgery.

The main inclusion criteria for the patients with COPD were a significant history of tobacco smoking and spirometry-defined COPD diagnosis according to GOLD guidelines. Exclusion criteria were any atopy or allergic disease as well as any non-COPD airway-related disease (including any physician-diagnosed asthma and upper airway conditions such as rhinitis or nasal polyposis). The smoking or never-smoking control patients had no history of respiratory disease and no symptoms of infections for at least 4 weeks prior to surgery. All clinical procedures were performed at the Department of Thoracic Surgery, Department Respiratory Medicine and Allergology, Lund/Skåne University Hospital, Sweden and the Department of Respiratory Medicine, Amsterdam Medical Center (AMC), Amsterdam, Netherlands All

procedures were approved by the local Swedish Research Ethics Committee in Lund, Sweden (Dnr 2015/891) and the local Medical Ethics Committee of the AMC (METC 2015_021), Amsterdam. All patients signed informed consent to participate in the study. The clinical sampling protocols and tissue processing to paraffin blocks were identical between the Lund and Amsterdam sites.

After surgery, excised large lung regions were immediately dissected into smaller lung tissue sections that were immersed in standard 4% buffered paraformaldehyde. This procedure allows a rapid cross-linking of proteins and RNA by the fixative and minimize any post-surgical transcript or protein expression alteration.

After fixation for 24 hours, the tissues were trimmed into smaller blocks, dehydrated, and embedded into paraffin blocks containing multiple anatomic regions: alveolar parenchyma, bronchi, bronchioles, and pulmonary vessels. To avoid tentative mechanical crush damages or artefactual molecular alterations at the cut sample edges, the immediate outer border of the subsequent 3 um paraffin sections was excluded from the histological analyses below.

Single-double immunohistochemistry (IHC)

Prior to IHC, heat-induced epitope retrieval (HIER) was performed in a pretreatment module (PT-link, Dako Cytomation, Glostrup, Denmark) with pH 6 or 9 buffer antigen retrieval buffer (Agilent Technologies, Santa Clara, CA, US). All IHC procedures were performed using an automated immunohistochemistry robot (Autostainer LINK, Dako) with EnVision™ immunohistochemistry and detection system (Dako). Appropriate positive and negative control tests, for example using isotype control antibodies was performed to rule our non-specific binding of the secondary antibody and the detection reagents.

<u>IL-33 single immunohistochemistry:</u>

HIER treated sections were blocked with endogenous enzyme block (EnVision™ FLEX Peroxidase-Blocking Reagent, Dako) before incubation with an anti-IL-33 antibody (clone Nessy-1, see table E1) and detection with anti-rabbit/mouse-HRP (K8010 Dako). Finally, the signal / immunoreactivity was developed using diaminobenzidine (DAB) chromogen, resulting in a dark brown insoluble staining. Hematoxylin was used as counterstain (blue nuclei) and the section was thereafter mounted with Pertex mounting medium (Leica Microsystems, Wetzlar, Germany). The specificity of the immunostaining was confirmed by negative staining with isotype control antibodies.

In situ hybridization (ISH)

Paraffin sections were deparaffinized, incubated with endogenous enzyme block, boiled in target retrieval solution, and treated with protease, followed by target probe hybridization. The human housekeeping gene PPIB was used as positive control gene and DapB (soil bacteria mRNA) as negative mRNA control probe. The target mRNA was amplified using a series of amplification solutions and detected with Vina Green and Fast Red chromogen (bright field) or tyramide fluorophores (Fluorescence ISH, Biotium, Fremont, CA, USA). Nuclei staining was used as background counter stain and visualized with the DNA binding probe Hoechst 33342 (Sigma-Aldrich, St. Louis, MO, USA). Finally, the tissue sections were dehydrated and mounted under glass coverslips.

For full tissue / patient quantification (Figs 1-2), dual visualization of total ST2 mRNA together with IL-33 mRNA and initial single visualization of transcript variants of soluble (sST2) or membrane bound (ST2L) isoforms of ST2 was performed through double in situ hybridization using the RNAscope™ 2.5 HD assay kits (RED #322360 and Duplex #322430; Advanced Cell

Diagnostics, Hayward, CA, USA) for chromogenic ISH (CISH), according to the manufacturer's instructions.

In depth quantitative mapping of sST2 mRNA or ST2L mRNA on leukocytes and structural cells was performed by a 2-4-plex fluorescent in situ hybridization protocol (RNAscope^{TM} multiplex fluorescent reagent kit v2 #323100, Advanced Cell Diagnostics, according to manufacturer’s instructions (see Table E1 for mRNA probe details) followed by immunohistochemical visualization of leukocyte (including mast cells) and structural cell markers.

For in depth mapping of alveolar capillaries a 4-plex ISH with ST2L or sST2 (Cy3 channel) CD31/PECAM1 mRNA (FITC channel), the aerocyte marker EDNRB mRNA (Cy5) and the gCAp marker PTPRB mRNA (Cy7) was performed followed by multiplex IHC (see below).

Multiplex immunohistochemistry and combined in situ hybridization (mIHC-ISH)

For proper identification and quantification of ST2 and IL-33 expressing cells, quadruple ISH (see above) was integrated with a platform for multiplex (IHC) staining (Additive Multiplex Labelling Cytochemistry, AMLC Platform, Medetect AB, Sweden). AMLC uses the principle of cyclic immunohistochemistry for simultaneous visualization of multiple leukocytes, structural cell populations, and phenotype markers within single sections (1). In brief, IHC cycles with intermediate blockings and/or elution were performed in an automated histochemistry robot, and the accumulating staining pattern was assembled by microscope-based whole slide scanning and computer-based image handling and (Cell Community Viewer, 2023, Medetect AB). Apart from technical assay controls and negative and positive control tests, all used primary antibodies (outlined in Table E1) have been previously validated for IHC on FFPE paraffin sections (many are also approved for in vitro diagnostic use).

Quantitative computerized image analysis of marker expression

The accumulated series of IHC and ISH marker-specific high-resolution digital images were processed by purpose-built software (Cell Community Viewer, CCV, Medetect AB, Lund, Sweden) to digitally identify and assemble the marker-specific cell objects, generating high-resolution multiplex images. A separate tissue detection algorithm was used to segment out the background tissue area outline. For bulk marker quantification within tissue region of interests (ROIs), markers were quantified automatically and the total amount of marker-positive pixels for each marker was normalized to the total analyzed tissue area within the section (or pre-selected ROI). The data for such measurements was presented as ratio of marker positivity (% of the analyzed tissue area). Furthermore, by using an automated single cell object algorithm (CCV, Medetect AB, Sweden), individual cell objects were analyzed for marker expression in marker-defined cells. Single cells were segmented with an algorithm based on individual separated nuclei and cell type-specific marker distribution. Cell masks for marker-positive cells were then measured for content of all other markers within the marker series (numbers and intensity of immunoreactivity (IHC) or ISH mRNA probe dots). To analyze marker staining profiles within distinct tissue compartment, the epithelium, airway wall, pulmonary vessels, alveolar parenchyma, and lymphoid aggregates were delineated on blinded digitalized sections by manual cursor tracing (supported by an AI-based epithelium recognition tool).

For each patient, large sections containing bronchioles, pulmonary vessels, and alveolar parenchyma were analyzed from 2–3 separate lung regions. The cell density in each compartment was calculated as the number of cells / assessed tissue area. Alveolar parenchyma was defined as the alveolar tissue only (e.g., after small airways and larger vessels have been excluded from the analysis).

Spatial cluster plots

By using an automated cluster plot algorithm (Medetect AB, Sweden), the x,y coordinates for marker-positive individual cells were plotted on a tissue background image and color-coded according to the number of spatially neighboring cells within a radius of 200 pixels (corresponding to 50 um). sST2^{high} microenvironments were defined as areas where sST2^{+} cells had >30 other sST2 neighboring cell objects whereas the cut off for sST2^{low} areas was defines as <20 sST2^{+} neighboring objects.

Assessment of IL-1RL1 and IL-33 expression patterns from single cell RNA seq data

To analyze the expression of *IL1RL1* (ST2) and IL-33 in human lung cells, we utilized raw data from a previously published single-cell RNA sequencing (scRNA-seq) study (6). In total 75,000 cells from various lung tissue compartments were subjected to single cell RNA sequencing using the droplet-based approach from 10x Genomics as the major analysis platform. Whereas the cells were from resected lung tissues obtained from three patients undergoing lobectomy for lung tumors, only non-tumor tissue regions with a normal looking morphology were used for the analysis. We analyzed the expression of IL-33 and *IL1RL1* across selected cell clusters. The expression data for IL-33 and *IL1RL1* were collected and visualized, demonstrating expression in the following cell types: adventitial fibroblast, artery, basal cells, bronchial, capillary A (aerocytes), capillary intermediate, lipofibroblast, mast cells, and vein (Figure 6 C in the main manuscript). The IL-33 and *IL1RL1* expression values were extracted and presented as average log fold change (avg_logFC) and the percentage of cells expressing the respective gene within each cluster (pct_in_cluster).

To confirm our findings, we also utilized another single-cell RNA sequencing publication that provided a scRNA-seq dataset of 312,928 cells derived from human lung tissues (7). Information on IL-33 and *IL1RL1* expression across epithelial, stromal, endothelial, and immune cell types was obtained from pooled patient data. Among these were vascular endothelial cells, including alveolar capillary subtype A (characterized as aerocytes, aCAPs showing elevated expression of EDNRB and HPGD), and capillary subtype B (identified as general capillary cells, gCAPs), characterized by high expression of FCN3 and IL7R); Fig 6D in the main manuscript).

Statistical analysis

GraphPad Prism (version 10.0.0, GraphPad Software, Boston, Massachusetts, USA) and JMP® (Version 17, SAS Institute Inc., Cary, NC, 2023) were used for the statistical analyses. For each variable and group, we calculated mean, median, standard error, standard deviation, and interquartile range. We performed the non-parametric Mann-Whitney U test for comparison of two sample populations and Kruskal–Wallis test followed by the Dunn's post hoc test for comparing two or more independent sample groups. In addition, we performed the Spearman correlation test between variables; coefficients (r) were considered statistically significant at $p<0.05$.

REFERENCES

E1. Erjefalt JS, de Souza Xavier Costa N, Jonsson J, Cozzolino O, Dantas KC, Clausson CM, et al. Diffuse alveolar damage patterns reflect the immunological and molecular heterogeneity in fatal COVID-19. *EBioMedicine* 2022; 83: 104229.

E2. Jogdand P, Siddhuraj P, Mori M, Sanden C, Jonsson J, Walls AF, et al. Eosinophils, basophils and type 2 immune microenvironments in COPD-affected lung tissue. *Eur Respir J* 2020; 55.

E3. Hogg JC, Chu F, Utokaparch S, Woods R, Elliott WM, Buzatu L, et al. The nature of small-airway obstruction in chronic obstructive pulmonary disease. *N Engl J Med* 2004; 350: 2645-2653.

E4. Andersson CK, Mori M, Bjermer L, Lofdahl CG, Erjefalt JS. Alterations in lung mast cell populations in patients with chronic obstructive pulmonary disease. *Am J Respir Crit Care Med* 2010; 181: 206-217.

E5. Roos AB, Sanden C, Mori M, Bjermer L, Stampfli MR, Erjefalt JS. IL-17A Is Elevated in End-Stage Chronic Obstructive Pulmonary Disease and Contributes to Cigarette Smoke-induced Lymphoid Neogenesis. *Am J Respir Crit Care Med* 2015; 191: 1232-1241.

E6. Travaglini KJ, Nabhan AN, Penland L, Sinha R, Gillich A, Sit RV, et al. A molecular cell atlas of the human lung from single-cell RNA sequencing. *Nature* 2020; 587: 619-625.

E7. Adams TS, Schupp JC, Poli S, Ayaub EA, Neumark N, Ahangari F, et al. Single-cell RNA-seq reveals ectopic and aberrant lung-resident cell populations in idiopathic pulmonary fibrosis. *Sci Adv* 2020; 6: eaba1983.

Table E1. Primary antibodies and mRNA probes used in single-multiplex immunohistochemistry and in situ hybridization

Antibody	Manufacturer, cat no.	Dilution 1:x	Target
Immune markers, IHC			
CD45	Dako*, M0701	50	Leucocyte common antigen
CD3	Dako, A0452	100	T lymphocytes
CD8	Dako, M10301-2	1000	CD8+ T lymphocytes
CD20	LS-Bio, LS-B2605	100	B lymphocytes
CD68	Dako, M087629	200	Monocytes and macrophages
CD163	Leica, NCL-L-CD163	80	Monocytes and macrophages
MPO	Dako, A0398	10000	Neutrophil myeloperoxidase
CD11c	Leica, NCL-L-CD11c-563	50	Dendritic cells, macrophages, NK
Fascin	Dako, M3567	100	Lymphoid tissue-associated DCs
ECP	Diagnostics development	800	Eosinophils
Tryptase	Millipore, MAB1222A	8000	Mast cells
Chymase	Atlas, HPA052634	8000	Mast cells
IL-33	Enzo, ALX-804-840-c100	900	Interleukin 33
ST2	AZ, in house	n/a	IL1RL1, IL-33 receptor
Structural markers, IHC			
Pro-SPC	LS-Bio, LS-B9161	3000	Prosurfactant protein C, Alveolar type II cells
RAGE	Abcam, ab216329	6000	Alveolar type I cells
Cytokeratin	Leica, NCL-L-AE1/AE3	300	Broad spectrum cytokeratin, epithelial cells
CD34	Dako, M7165	100	Endothelial cells, hematopoietic cells
CD146	GeneTex, GTX01919	50	Endothelial cells, pericytes
SMA	Dako, M085129	300	Smooth muscle actin
Vimentin	Abcam, ab45939	800	Fibroblast intermediate filament
mRNA Probes, ISH			
IL-33	ACD Bio, 400111	n/a	IL-33
IL1RL1	ACD Bio, 603491	n/a	Pan-ST2 (detects all ST2 transcript variants)
IL1RL1-O2[#]	ACD Bio, 569671	n/a	Soluble ST2 / sST2
IL1RL1-O3[§]	ACD Bio, 569681	n/a	Membrane anchored receptor for IL-33, ST2L, ST2L
PECAM-01	ACD Bio, 487381	n/a	PECAM1/CD31, endothelial cells
PTPRB	ACD Bio, 588141	n/a	gCap, general capillary cells
EDNRB	ACD Bio, 528301	n/a	aCap, aerocytes (capillary cell phenotype)

*ISH: in situ hybridization, IHC: immunohistochemistry, n/a: not applicable. * Dako / Agilent. [#]Detects IL1RL1 sST2 + NM_001282408.1 (transcript variant 3) and NR_104167.1 (transcript variant 4, which is non-coding). Does not detect ST2L. [§]Only detects ST2L / ST2L transcripts*

Table E2. Characteristics of the study subjects used for in depth analysis

Characteristic	Controls (smokers and non-smokers)	GOLD IV COPD
Subjects, *n*	8	8
Gender, men/women	2/6	4/4
Age, years	36 (68-81)	63 (52-66)
BMI	21 (25-53)	25 (17-28)
Smoking history, pack-years	22 (1-40)	45 (12-50)
Smoking status,		
never/ex-smokers/current	1/6/1	0/8/0
FEV1, L	2.0 (1.6-2.7)	0.8 (0.5-0.9)
FEV1, % of predicted	84 (59-98)	24 (22-28)
FEV1/VC	0.7 (0.7-0.8)	0.3 (0.2-0.4)
DLCO, % of predicted	72 (60-109)	28 (20-41)
Medication		
Inhaled β2 agonists		
Short acting (yes/no/unknown)	0/8/0	3/4/1*
Long acting (yes/no/unknown)	0/8/0	4/3/1*
Inhaled anticholinergics		
Short acting (yes/no/unknown)	0/8/0	3/4/1*
Long acting (yes/no/unknown)	0/8/0	6/1/1*
Corticosteroids		
Inhaled (yes/no/unknown)	0/8/0	7/0/1*
Oral (yes/no/unknown)	0/8/0	1/6/1*

Values are median (range) or n. COPD, chronic obstructive pulmonary disease; FEV1; forced expiratory volume in 1 s; VC, vital capacity; GOLD, Global Initiative for Chronic Obstructive Lung Disease. The patients used for the in-depth histology-based single-cel analysis of IL-33 and ST2 expressing cells were the subcategory of control subjects and GOLD IV patients from Lund /Skane University Hospital.

**One patient with incomplete medical treatment history.*

Online Supplement Figures

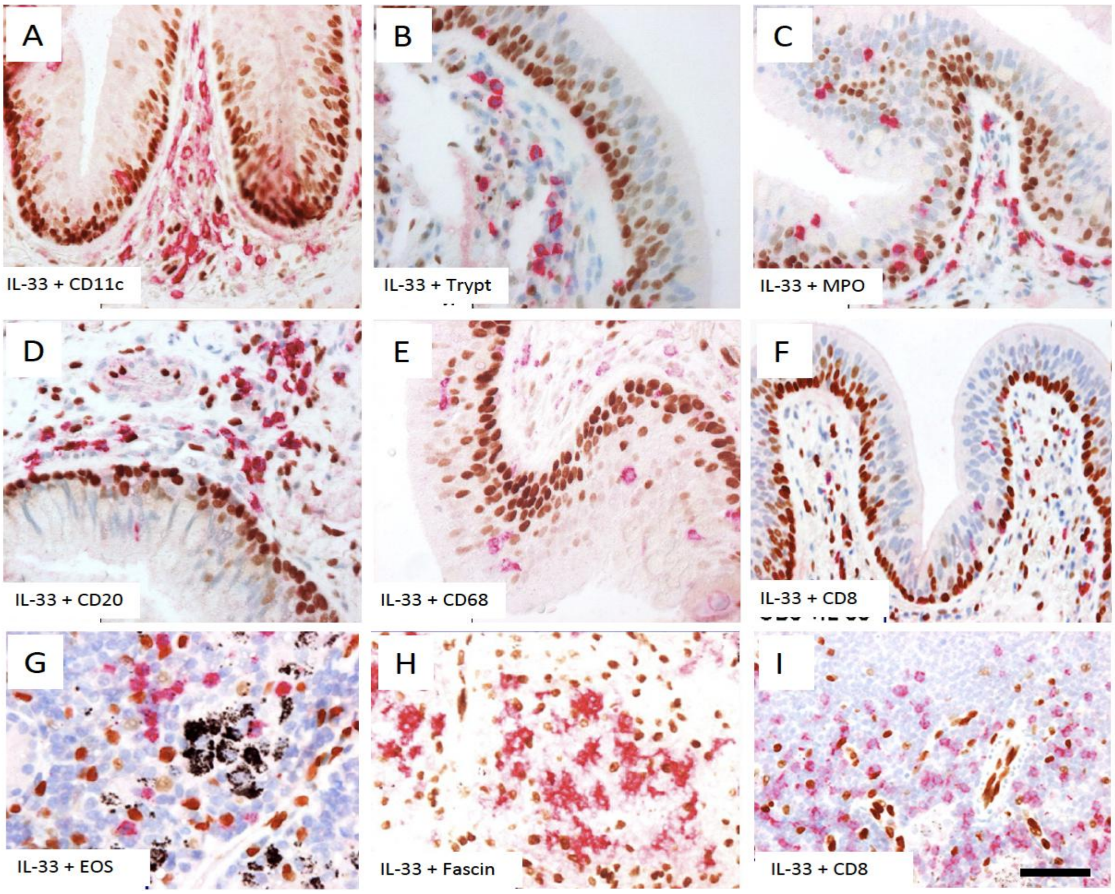

Figure E1. **(A-I)** Double immunohistochemical staining with pairwise examples of non-overlapping IL-33 and leukocyte marker immunoreactivity. IL-33 is detected with brown DAB chromogen and leukocyte markers with Fast Red chromogen. Panels A-F are from COPD bronchioles / small airways, whereas G-I are from lung lymphoid tissue. Trypt = mast cell tryptase, EOS = staining for eosinophil cationic protein, MPO = myeloperoxidase (mainly expressed by neutrophils).

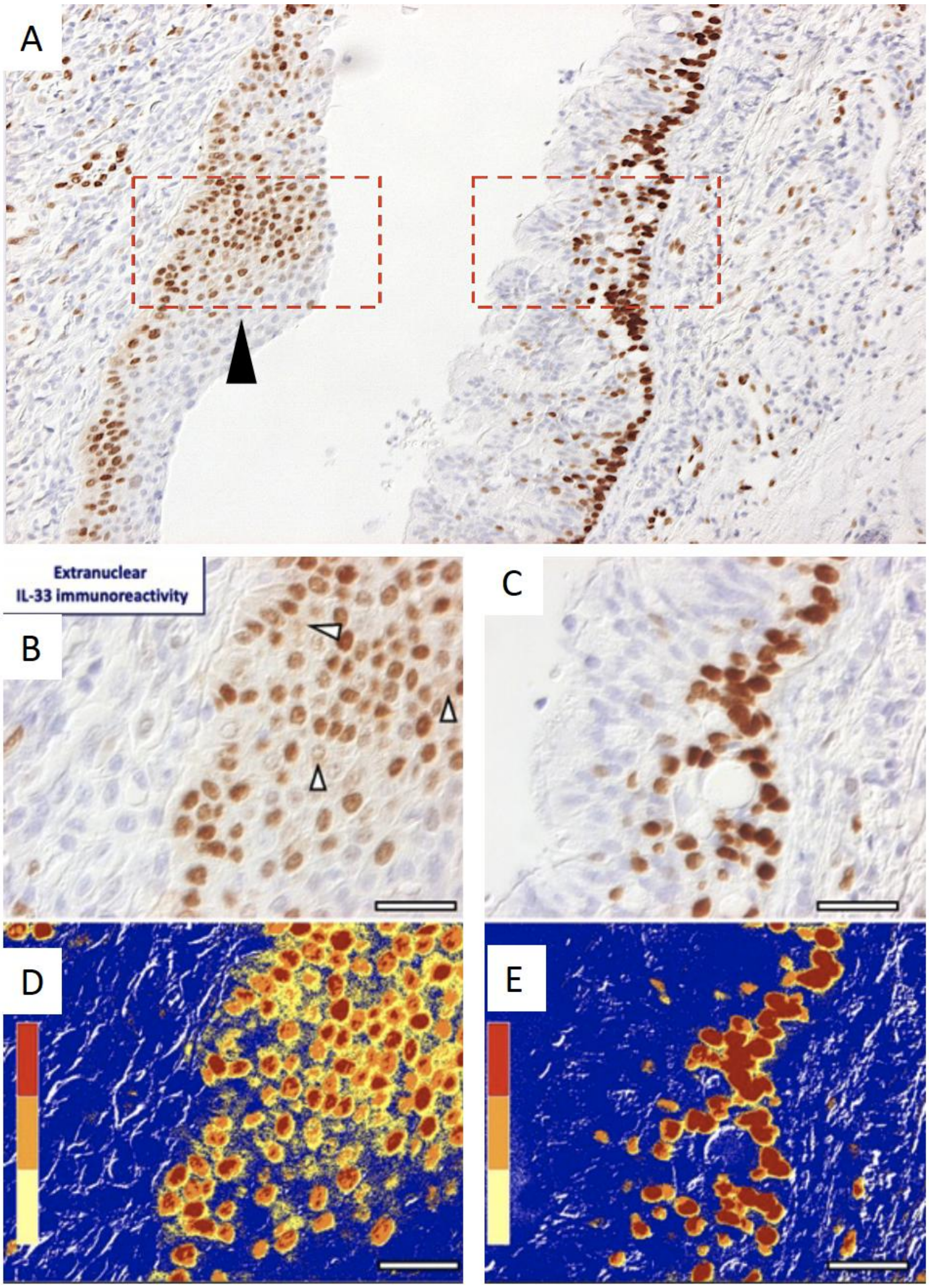

Figure E2. **(A)** A longitudinally sectioned bronchiole from a GOLD 4 patient exemplifying a patchy focal metaplastic epithelium (left rectangle) and opposing normal pseudostratified epithelium (right rectangle). (B-E) Zoomed in views of the rectangle areas to visualize partial loss of IL-33 nuclear protein and extranuclear IL-33 in the metaplastic region. In panels D-E computerized image analysis has been used to segment out IL-33 immunoreactivity and display color-coded staining intensity.

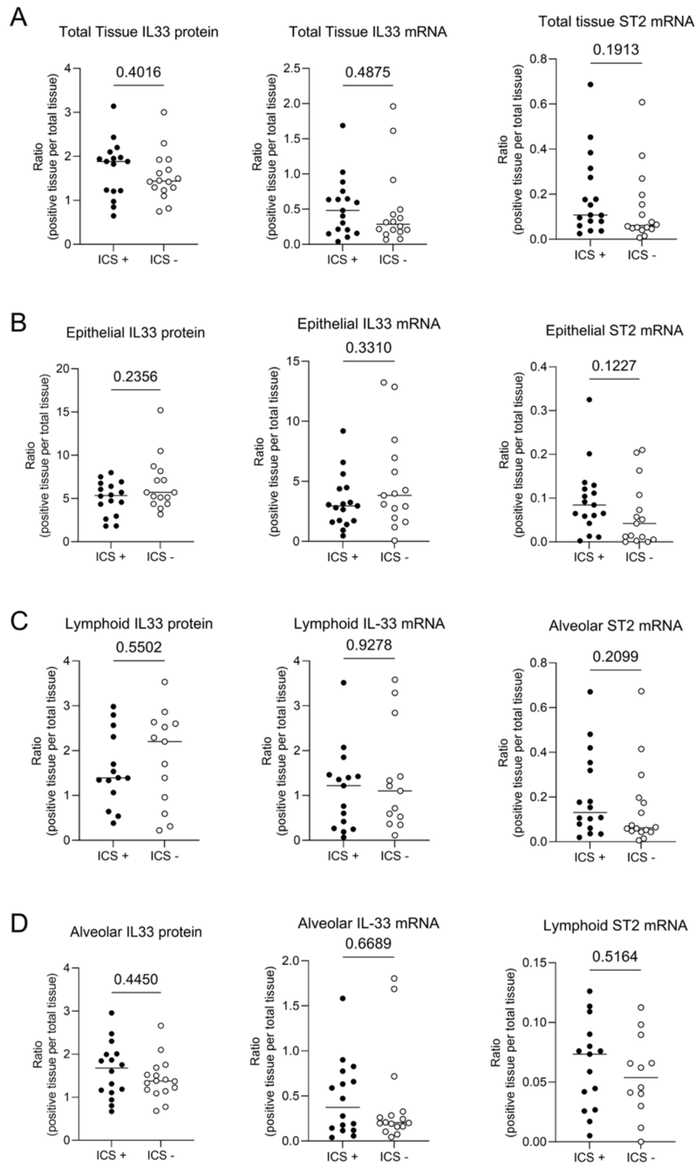

Figure E3. Compartment-specific expression of IL-33 and ST2 in lung tissue from ICS-treated (ICS+) and untreated (ICS–) subjects. Immunohistochemistry and in situ hybridization were performed to assess IL-33 protein, IL-33 mRNA, and ST2 mRNA expression across distinct lung tissue compartments. Quantification is shown for (A) total tissue, (B) epithelial, (C) lymphoid, and (D) alveolar regions. Expression is presented as the ratio of positive tissue area per total tissue area. Each dot represents an individual subject (black circles: ICS+; open circles: ICS–). Horizontal bars indicate median values. P values were calculated using the Mann–Whitney U test.

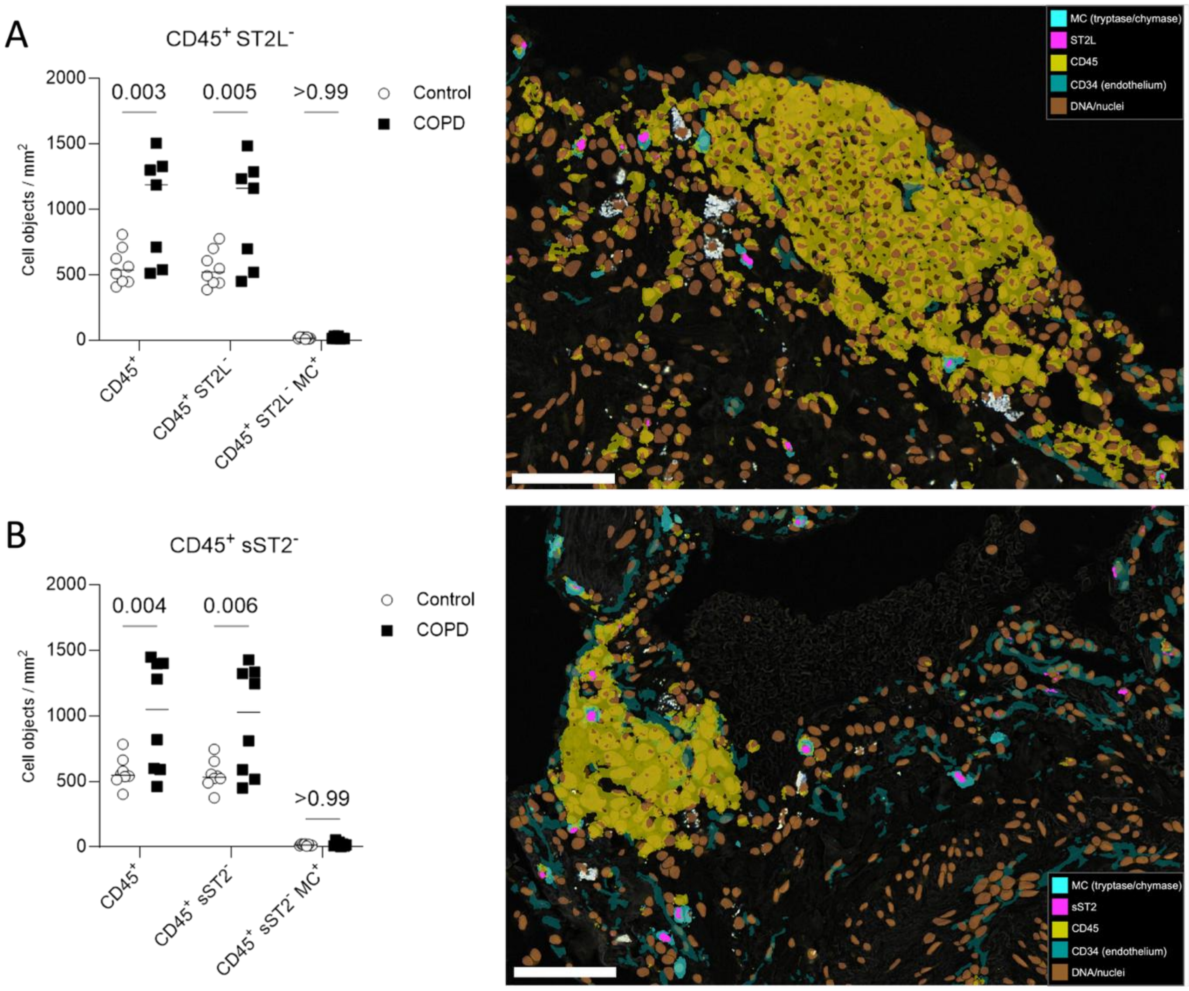

Figure E4. CD45⁺ ST2⁻ immune cells. Quantification of total CD45$^+$, CD45$^+$ ST2L$^-$, and CD45$^+$ ST2L$^-$ MC$^+$ cells (A, left). Visualization of tryptase/chymase-identified mast cells (turquoise), ST2L mRNA (pink) and CD45 (yellow) in the alveolar parenchyma (A, right). Quantification of total CD45$^+$, CD45$^+$ sST2$^-$, and CD45$^+$ sST2$^-$ MC$^+$ cells (B, left). Visualization of tryptase/chymase-identified mast cells (turquoise), sST2 mRNA (pink) and CD45 (yellow) in the alveolar parenchyma (B, right). Control shown as open circles and COPD in filled squares. Horizontal bars represent median values. P values by Mann–Whitney U test. Scale bar=100 μm.

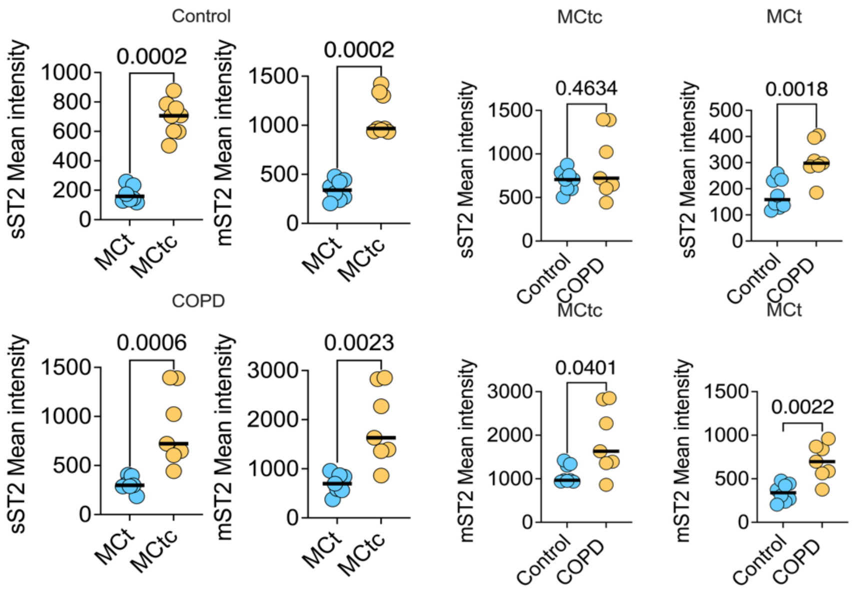

Figure E5. (A) Comparisons between sST2 and ST2 mRNA expression in MCT and MCTC mast cell subtypes in total lung tissue from control subjects and COPD patients. (B) Comparisons between control and COPD lungs regarding MC$_T$ and MC$_{TC}$ mast cell expression of sST2 and ST2L. Statistical significance between groups was tested using Kruskal Wallis with Dunn's multiple comparison test.

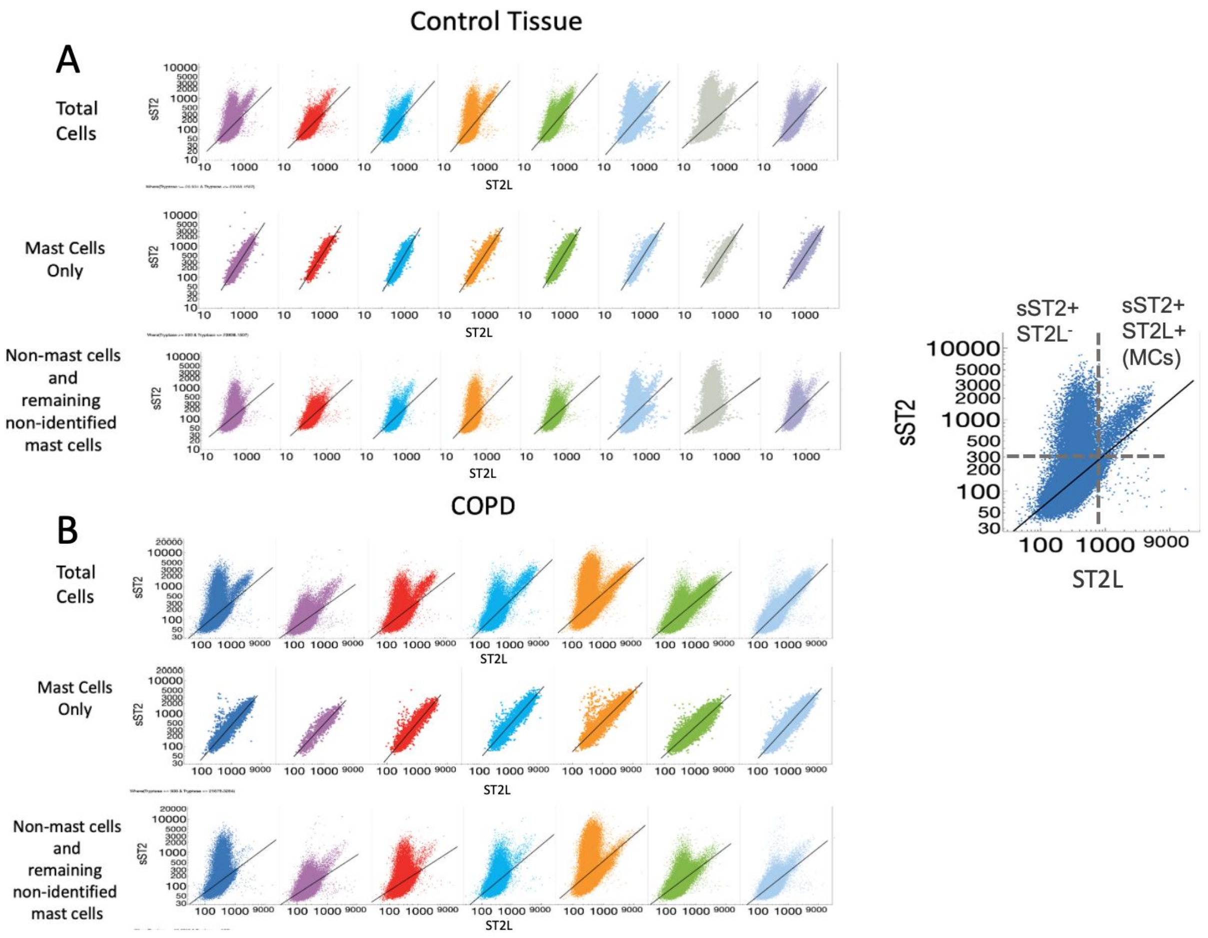

Figure E6. Histology-based single cell analysis with sST2 and ST2L expression intensities in alveolar parenchymal cells from individual control subject (A) and COPD patients (B). The mRNA staining intensity was calculated by computerized image analysis. Data are shown for > 2000 cells / patient. Y and X axes shows ST2 and ST2L mRNA ISH-based staining intensity.

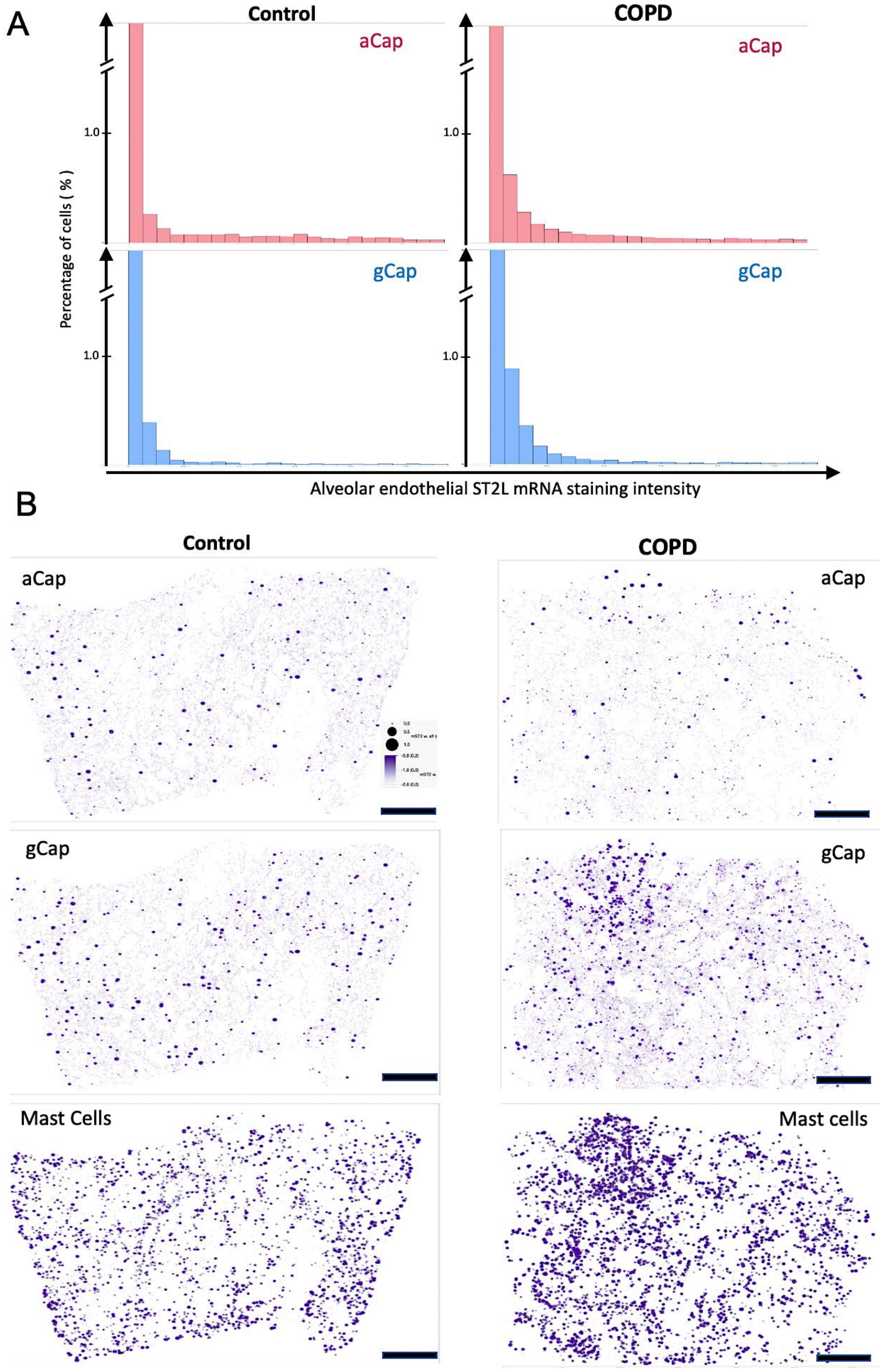

Figure E7. **(A)** Histogram showing the proportions (%) of pre-filtered CD31 /PECAM-positive alveolar endothelial cells across intensity levels ST2L mRNA, as measured through histology-based single cell analysis. Note that the first left bar corresponds to absent expression and that this category represent most of the cells. (B) Examples of spatial distribution graphs where x,y coordinates for aCap and gCAps (identified by CD31 /PECAM+cells with EDNRB and PTPRB, respectively) and mast cells have been marked with circles color- and size-coded for their ST2L mRNA expression (higher expression = larger and darker dots). Scale bars = 4 mm.

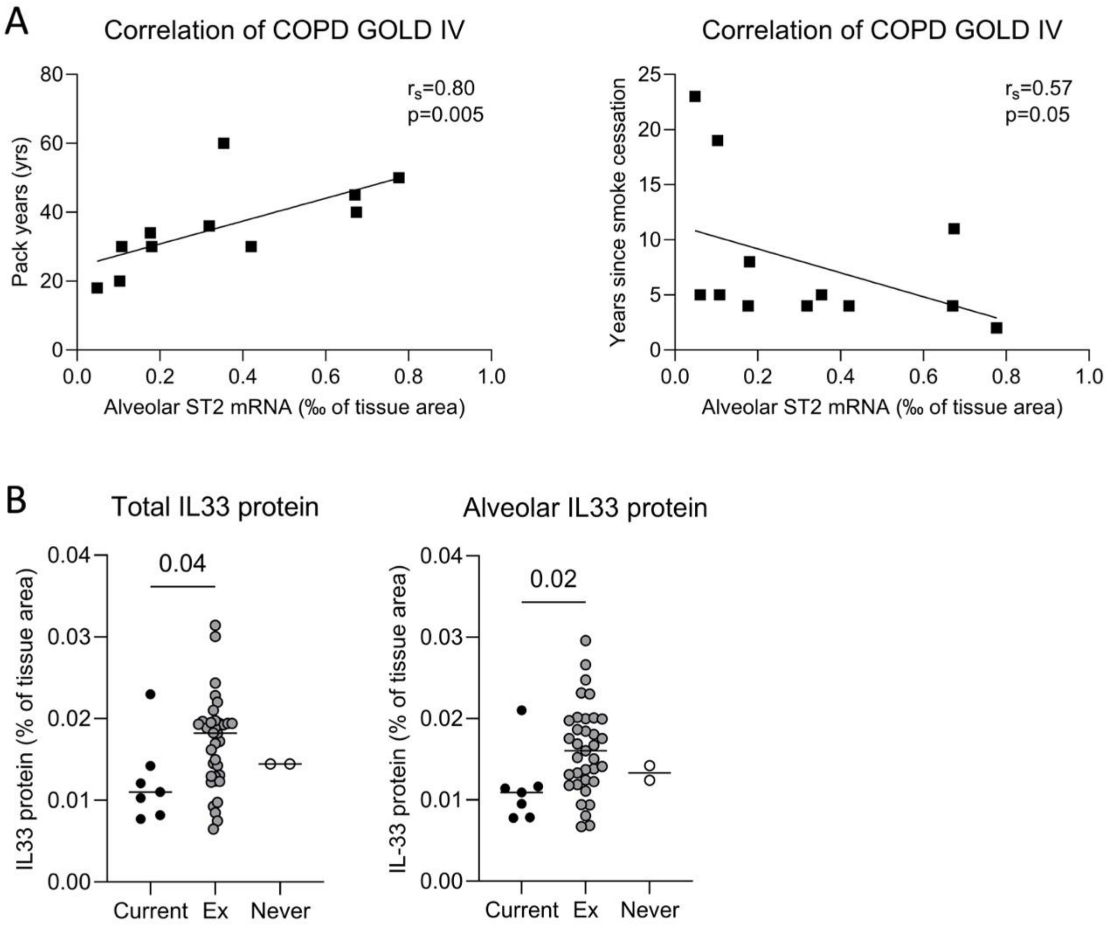

Figure E8. Relationship of IL-33/ST2 expression with smoking history and COPD status. (A) Correlation analyses in COPD GOLD IV patients showing (left) a positive association between alveolar ST2 mRNA expression (% of tissue area) and cumulative smoking exposure (pack years) and (right) a negative association between alveolar ST2 mRNA expression and years since smoking cessation. Spearman's rank correlation coefficients (r_s) and p values are indicated. (B) IL-33 protein expression in (left) total tissue and (right) alveolar compartment compared among current smokers, ex-smokers (Ex), and never-smokers. Expression is quantified as the percentage of tissue area positive for IL-33 protein. Each symbol represents an individual subject; horizontal lines denote medians. P values were calculated using the Mann–Whitney U test.
